# Four new indole alkaloids from *Plantago asiatica*

**DOI:** 10.1007/s13659-012-0082-4

**Published:** 2012-12-20

**Authors:** Zhong-Hua Gao, Ling-Mei Kong, Xi-Sheng Zou, Yi-Ming Shi, Shan-Zhai Shang, Huai-Rong Luo, Cheng-Qin Liang, Xiao-Nian Li, Yan Li, Xue Du, Wei-Lie Xiao, Han-Dong Sun

**Affiliations:** 1 State Key Laboratory of Phytochemistry and Plant Resources in West China, Kunming Institute of Botany, Chinese Academy of Sciences, Kunming, 650201 Yunnan China; 2 University of Chinese Academy of Sciences, Beijing, 100049 China

**Keywords:** *Plantago asiatica*, indole alkaloid, AChE inhibitory activity, cytotoxic activity

## Abstract

Four new indole alkaloids, plasiaticines A-D (**1**–**4**), together with two known ones, were isolated from the seeds of *Plantago asiatica*. The structures of the new compounds were elucidated on the basis of comprehensive analysis of spectroscopic data. All compounds were tested for their cytotoxic activity, and all compounds except **4** were tested for their acetylcholinesterase (AChE) inhibitory activities.

**Electronic Supplementary Material:**

Supplementary material is available for this article at 10.1007/s13659-012-0082-4 and is accessible for authorized users.

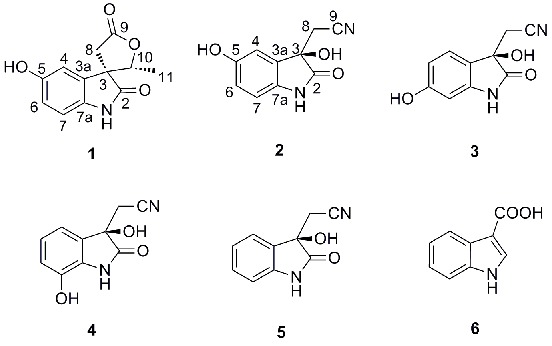

## Introduction

Most species of *Plantago* genus are perennial herbs widely distributed in China, and the aerial parts of many *Plantago* species have been used as diuretic,[1] expectorant,[2] anti-inflammatory agent[3] in folk medicine for a long time. Phytochemical research found that this genus produced polysaccharides,[4,5] flavonoids,[6–8] caffeic acid glycoside esters,[9,10] phenylethanoid glycosides,[5,10–12] iridoid glucosides,[13–17] and triterpene acids,[8,18,19] which showed various bioactivities, such as anti-inflammatory,[20] antibacterial,[21,22] antioxidant,[11,23] immunesuppressant,[24] analgesic,[25] and antiallergic activity.[7,26] The seeds and whole plants of *P. asiatica* are recorded in Pharmacopoeia of People's Republic of China.[27] Previous studies on *P. asiatica* have led to the identification of several kinds of components, including polysaccharides,[4,5] phenylethanoid glycosides,[5,10–12] and phenolic constituents.[28] Polysaccharides from the seeds of *P. asiatica* were found to enhance the immune function of the immunosuppressant mice in the previous study.[29] Motivated by a search for bioactive metabolites from seeds of this plant, an investigation of the chemical constituents was carried out. As a result, four new indole alkaloids, plasiaticines A–D (**1**–**4**), along with two analogues (**5** and **6**), were isolated. Among them, compound **5** was firstly isolated from natural source. In this paper, we report the isolation, structure determination, cytotoxic activity and AChE inhibitory activity of these compounds.

*To whom correspondence should be addressed. E-mail: xwl@mail.kib.ac.cn (W.L. Xiao); hdsun@mail.kib.ac.cn (H.D. Sun)

**1**

**2**

**3**

**4**

**5**

**6**

## Results and Discussion

A 70% aqueous acetone extract of the seeds of *P. asiatica* was partitioned between EtOAc and $H_2O$. The EtOAc solubles were dried and subjected to silica gel, Sephadex LH-20 and Lichroprep RP-18 gel column chromatography (CC) and semipreparative HPLC to afford four new compounds, plasiaticines A–D (**1**–**4**), together with two known ones, (+)-(*R*)-3-cyanomethyl-3-hydroxyoxindole (**5**),[30,31] and indolyl-3-carboxylic acid (**6**).[32]

Compound **1** was obtained as colorless oil and had the molecular formula of $C_{12}H_{11}NO_4$ as determined by the analysis of its NMR spectroscopic data and verified by HREIMS (found *m/z* 233.0687, calcd for 233.0688), requiring eight degrees of unsaturation. The IR spectrum exhibited absorption bands for hydroxy (3423 $cm^{-1}$) and $\gamma$-lactone carbonyl (1789 $cm^{-1}$) groups and an aromatic ring (1499 and 1475 $cm^{-1}$). The UV spectrum displayed absorption maximum at 309, 261 and

207 nm. These characteristic IR and UV absorptions indicated **1** was an oxindole-type compound.[33,34] The $^{1}$H NMR spectroscopic data (Table 1) showed one set of 1,3,4-trisubstituted benzene protons, one oxygenated methine group connecting with a methyl, and two methylene protons neighboring a carbonyl group. The $^{13}$C NMR and DEPT spectra (Table 2) showed one methyl, one methylene, four methines (one oxygenated and three olefinic), and six quarternary carbons (two carbonyls and three olefinic ones). Apart from an oxindole core and a carbonyl, the remaining one degree of unsaturation of **1** was determined to be a lactone skeleton. One pair of methylene protons [$\delta_H$ 3.06 (d, $J$ = 17.1 Hz, H-8), 2.76 (d, $J$ = 17.1 Hz, H-8)] presenting AB system suggested that the C-3 to be a spiro-quarternary carbon. This was further confirmed by HMBC correlations of of H-8 with C-2, C-3, C-9 and C-10, and of H-11 with C-3 and C-10 (Figure **1**), together with $^{1}$H-$^{1}$H COSY correlation of H-10/H-11 (Figure **1**). These evidence suggested that compound **1** might have the same structure core as that of coixspirolactam B.[35] Comparison of the NMR data of **1** with those of coixspirolactam B showed that the difference could be rationalized to the replacement of a hydroxy group substituted at C-5. This was further confirmed by HMBC correlations of H-4 with C-3a, C-5 and C-7a, of H-6 with C-4 and C-5, of H-7 with C-3a, C-5 and C-7a (Figure **1**), together with $^{1}$H-$^{1}$H COSY correlations of H-6/H-7 and H-10/H-11 (Figure **1**).

**Table 1.** $^{1}$**H NMR data of 1–4 (**$\delta$ **in ppm,** $J$ **in Hz)**

| pos. | 1[a] | 2[b] | 3[c] | 4[d] |
|---|---|---|---|---|
| 1 | 8.26 (br. s) | 11.77 (br. s) | 11.86 (br. s) | 12.69 (br. s) |
| 4 | 7.07 (d, 1.8) | 7.93 (br. s) | 7.90 (d, 8.1) | 7.63 (d, 7.2) |
| 5 | | | 6.90 (dd, 8.1, 1.6) | 7.07 (t, 7.8) |
| 6 | 6.75 (br. dd, 8.4, 1.8) | 6.94 (d, 7.0) | | 7.17 (d, 8.4) |
| 7 | 6.79 (d, 8.4) | 7.15 (d, 7.0) | 6.83 (d, 1.6) | |
| 8a | 3.06 (d, 17.1) | 3.61 (d, 16.4) | 3.60 (d, 16.4) | 3.64 (d, 16.8) |
| 8b | 2.76 (d, 17.1) | 3.30 (d, 16.4) | 3.20 (d, 16.4) | 3.30 (d, 16.8) |
| 10 | 4.81 (q, 6.4) | | | |
| 11 | 1.22 (d, 6.4) | | | |

[a]Recorded at 400 MHz in acetone-$d_6$; [b]Recorded at 400 MHz in pyridine-$d_5$; [c]Recorded at 500 MHz in pyridine-$d_5$; [d]Recorded at 600 MHz in pyridine-$d_5$.

**Table 2.** $^{13}$**C NMR data of 1–4 (**$\delta$ **in ppm)**

| pos. | 1[a] | 2[b] | 3[c] | 4[d] |
|---|---|---|---|---|
| 2 | 178.2 s | 178.7 s | 179.4 s | 179.3 s |
| 3 | 55.7 s | 73.8 s | 73.4 s | 74.5 s |
| 3a | 129.6 s | 132.5 s | 121.4 s | 133.0 s |
| 4 | 112.3 d | 113.4 d | 126.2 d | 116.2 d |
| 5 | 154.2 s | 155.0 s | 109.7 s | 123.7 d |
| 6 | 116.0 d | 117.2 d | 161.2 s | 118.3 d |
| 7 | 111.2 d | 111.6 d | 99.6 d | 144.0 s |
| 7a | 135.4 s | 134.5 s | 144.5 s | 131.5 s |
| 8 | 39.2 t | 27.8 t | 27.9 t | 28.2 t |
| 9 | 174.3 s | 117.9 s | 117.9 s | 118.3 s |
| 10 | 82.1 d | | | |
| 11 | 14.7 q | | | |

[a]Recorded at 100 MHz in acetone-$d_6$; [b]Recorded at 100 MHz in pyridine-$d_5$; [c]Recorded at 125 MHz in pyridine-$d_5$; [d]Recorded at 150 MHz in pyridine-$d_5$.

The relative configurations of **1** were determined by correlations of H-4/H-8a and H-4/H-10; and of H-8a/H-10 in ROESY experiment (Figure **2**), which showed that **1** also possessed the same relative configurations as those of coixspirolactam B.[35]

**1** **2**

**Figure 1.** Key HMBC (H⌒C) and $^{1}$H-$^{1}$H COSY (—) correlations of **1** and **2**

**1** **2**

**Figure 2.** Key ROESY (H⌒H) correlations of **1** and **2**

On the base of the relative configurations of compound **1** established by ROESY spectrum, the absolute configurations of **1** were determined by the theoretical calculation method of the ECD spectra by using time-dependent DFT (TDDFT) method in B3LYP/6-31G+(d,p) level. The calculated ECD curve for 3*S*,10*R* stereoisomer of **1** was similar to the experimental one (Figure 3). In addition, molecular orbital (MO) analysis of the dominant conformer **1b** with 64.7% population in MeOH gave us more information to understand the experimental ECD spectrum of **1**. The negative Cotton effect (CE) at 225 nm resulted from the negative rotatory strength at 214 nm involving a $\pi\rightarrow\pi^*$ electronic transition from MO61 to MO67 (Figure 4). Consequently, the absolute configuration of **1** was determined to be 3*S*,10*R*. Therefore, compound **1** was established as shown and named plasiaticine A.

The molecular formula of **2** was deduced as $C_{10}H_8N_2O_3$ from its HREIMS (*m/z* 204.0546, calcd for 204.0535) and $^{1}$H and $^{13}$C NMR data, corresponding to eight degrees of unsaturation. Comparisons of these characteristic IR and UV absorptions with those of **1** indicated that **2** was also an oxindole-type compound.[33,34] The NMR data (Tables 1 and 2) suggested that **2** had the same oxindole moiety as that in (+)-(*R*)-3-cyanomethyl-3-hydroxyoxindole (**5**).[30,31] The structural difference between **2** and **5** was an additional hydroxy at C-5 in **2**, which was further confirmed by the HMBC correlations (Figure **1**).

The calculated ECD curve for the 3*R* stereoisomer of **2** was compared to the experimental one, suggesting that the calculated ECD curve corresponded well with the

**Figure 3.** Experimental and calculated ECD spectra of **1**

**Figure 4.** The most important orbitals involved in the key transitions of the conformer **1b** at B3LYP/6-31+G(d,p) level in MeOH with PCM model experimental one except for a little red-shift in the calculated ECD spectrum (Figure 5). Furthermore, MO analysis of the dominant conformer **2e** with 70.2% population in MeOH afforded comprehension of the experimental ECD spectrum of **2**. The electronic transitions from MO53 to MO58 involving a $\pi \rightarrow \pi^*$ transition gave rise to the positive rotatory strength at 217 nm, which could be assigned to the experimental positive CE at 211 nm. The electronic transitions from MO51 to MO54 resulted in the negative rotatory strength at 239 nm, contributing to the negative CE at 238 nm. The positive rotatory strength at 260 nm resulted from the electronic transitions from MO53 to MO55 involving a $\pi \rightarrow \pi^*$ transition, which correlated to the weak positive CE at 267 nm (Figure 6). Thus, the absolute configuration of **2** was established as shown and named plasiaticine B.

Compounds **3** and **4** were both isolated as colorless oil and had the same molecular weight as **2**. Detailed comparison of the NMR spectra led to the conclusion that the only difference among compounds **2**, **3** and **4** was the location of the hydroxy group on the aromatic ring. In compound **3**, the hydroxy group was substituted at C-6, which could be elucidated by $^1$H-$^1$H COSY correlation of H-4/H-5, and by HMBC correlations of H-4 with C-3, C-5, C-6, C-7a; of H-5 with C-3a, C-4, and C-6; and of H-7 with C-3a, C-5, C-6, and C-7a. In compound **4**, the hydroxy was located at C-7. This could be further confirmed by $^1$H-$^1$H COSY correlations of H-4/H-5/H-6, and by HMBC correlations from H-4 to C-3, C-6 and C-7a; from H-5 to C-3a and C-7; and from H-6 to C-4, C-5 and C-7. Their absolute structures were also determined by analysis of their CD spectra. The CD spectra of **3** and **4** were both consistent with that of **2** (Figures 24S and 32S in Electronic Supplementary Material). This evidence determined the absolute configuration of C-3 in both **3** and **4** were also determined to be *R*, as well.

**Figure 5.** Experimental and calculated ECD spectra of **1**

All compounds were assayed for their cytotoxicity against the HL-60, SMMC-7721, A-549, MCF-7, and SW-480 human tumor cell lines by the MTS method with cisplatin and taxol as positive controls.[36] However, no compound showed significant activity with $IC_{50}$ more than 40 $\mu$M. Compounds **1**–**3**, **5** and **6** were evaluated for their inhibitory activities against AChE, with tacrine as a positive control.[37] None of them showed inhibitory activity against AChE with the inhibition ratio less than 60%. Compound **4** was not further tested for its AChE inhibitory activity due to the limited amount available.

## Experimental Section

**General Experimental Procedures.** Optical rotations were measured with a JASCO DIP-370 digital polarimeter. UV spectra were obtained using a Shimadzu UV-2401A spectrophotometer. A BioRad FtS-135 spectrophotometer was used for scanning IR spectroscopy with KBr pellets, whereas CD spectra were recorded on a JASCO J-810 spectropolarimeter. 1D and 2D NMR spectra were recorded on Bruker AM-400, DRX-500 and Bruker Avance III-600MHz spectrometers. Unless otherwise specified, chemical shifts ($\delta$) were expressed in ppm with reference to the solvent signals. High-resolution electron impact mass spectra (HREIMS) were performed on a VG Autospec-3000 spectrometer under 70 eV. Column chromatography was performed using silica gel (80–100 and 200–300 mesh, Qingdao Marine Chemical, Inc., Qingdao, China). Semi-preparative HPLC was performed on an Agilent 1100 liquid chromatograph with a Zorbax SB-$C_{18}$, 9.4 mm × 25 cm, column. Fractions were monitored by TLC and spots were visualized by heating the silica gel plates sprayed with 10% $H_2SO_4$ in EtOH.

**Figure 6.** The most important orbitals involved in the key transitions of the conformer **2e** at B3LYP/6-31+G(d,p) level in MeOH with PCM model

**Plant Material.** The seeds of *P. asiatica* were purchased from Juhuacun Traditional Chinese Medicine Market, Kunming, Yunnan Province, China, in August 2011. A voucher specimen (No. KIB 2011-08-11) has been deposited at the State Key Laboratory of Phytochemistry and Plant Resources in West China, Kunming Institute of Botany, Chinese Academy of Sciences.

**Extraction and Isolation.** The air dried seeds of *P. asiatica* (10 kg) were extracted with 70% aqueous acetone (3 × 30 L, three days each) at room temperature. The solvent was removed *in vacuo* to afford a crude extract, which was dissolved in $H_2O$, and then extracted successively with EtOAc (1:1) and *n*-BuOH for three times, respectively. The EtOAc-soluble part (285 g) was subjected to silica gel column chromatography (silica gel, 80–100 mesh, $CHCl_3/Me_2CO$, 1:0, 9:1, 8:2, 7:3, 6:4 and 0:1 ) to afford fractions A–E. Fraction C was chromatographed on RP-18 gel and eluted with $MeOH/H_2O$ (30/70–100/0) to give five subfractions C1–C5. Subfraction C1 was further purified with repeatedly silica gel (200–300 mesh, petroleum ether/acetone), Sephadex LH-20 (MeOH) and followed by semipreparative HPLC ($MeOH/H_2O$, 65/35) to afford compounds **1** (8 mg) and **6** (12 mg). Subfraction C3 was purified by repeated chromatography on silica gel and then on semipreparative HPLC ($MeOH/H_2O$, 60/40) to give compounds **2** (12 mg), **3** (11 mg), **4** (3 mg), and **5** (11 mg).

**Plasiaticine A (1):** colorless oil; $[\alpha]^{23}_{D}$ − 14.3 (*c* 0.08, MeOH); UV (MeOH) $\lambda_{max}$ (log $\varepsilon$) 207 (3.80), 261 (3.42), and 309 (2.84) nm; IR (KBr) $\nu_{max}$ 3423, 1789, 1766, 1704, 1499, 1475, 1201 $cm^{-1}$; $^{1}H$ and $^{13}C$ NMR data, see Tables 1 and 2; positive ESIMS *m/z* 256 (100) $[M + Na]^+$; HREIMS *m/z* 233.0687 $[M]^+$ (calcd for $C_{12}H_{11}NO_4$, 233.0688).

**Plasiaticine B (2):** colorless oil; $[\alpha]^{23}_{D}$ + 18.1 (*c* 0.13, MeOH); UV (MeOH) $\lambda_{max}$ (log $\varepsilon$) 208 (3.34), 264 (2.92), and 315 (2.31) nm; IR (KBr) $\nu_{max}$ 3425, 2262, 1721, 1628, 1478, 1202 $cm^{-1}$; $^{1}H$ and $^{13}C$ NMR data, see Tables 1 and 2; positive ESIMS *m/z* 227 (100) $[M + Na]^+$; HREIMS *m/z* 204.0546 $[M]^+$ (calcd for $C_{10}H_8N_2O_3$, 204.0535).

**Plasiaticine C (3):** colorless oil; $[\alpha]^{23}_{D}$ + 88.7 (*c* 0.10, MeOH); UV (MeOH) $\lambda_{max}$ (log $\varepsilon$) 216 (3.81), and 271 (2.93) nm; IR (KBr) $\nu_{max}$ 3428, 2260, 1725, 1632, 1472, 1117 $cm^{-1}$; $^{1}H$ and $^{13}C$ NMR data, see Tables 1 and 2; EIMS *m/z* 204 $[M]^+$; HREIMS *m/z* 204.0506 $[M]^+$ (calcd for $C_{10}H_8N_2O_3$, 204.0535).

**Plasiaticine D (4):** colorless oil; $[\alpha]^{25}_{D}$ + 5.2 (*c* 0.20, MeOH); UV (MeOH) $\lambda_{max}$ (log $\varepsilon$) 214 (3.32), and 306 (2.57) nm; IR (KBr) $\nu_{max}$ 3443, 2263, 1721, 1631, 1492, 1384 $cm^{-1}$; $^{1}H$ and $^{13}C$ NMR data, see Tables 1 and 2; EIMS *m/z* 204 (100) $[M]^+$; HREIMS *m/z* 204.0538 $[M]^+$ (calcd for $C_{10}H_8N_2O_3$, 204.0535).

**ECD Calculation.** The theoretical calculations of compounds **1** and **2** were performed using Gaussian 09.[38] Conformational analysis was initially carried out using Maestro7.5 conformational searching, together with the OPLS_2005 molecular mechanics methods. The optimized conformation geometries, thermodynamic parameters, and populations of all conformations were provided in the Electronic Supplementary Material (Figures 8S and 16S, and Tables 1S to 4S, and 5S to 8S). Then the OPLS_2005 conformers were then optimized at B3LYP/6-31G(d) level. The room temperature equilibrium populations were calculated according to Boltzmann distribution law. The ECD was performed at B3LYP/6-31G+(d,p) level in MeOH with PCM model. The ECD spectra of compounds **1** and **2** were obtained by weighing the Boltzmann distribution rate of each geometric conformation.

**ECD Simulation.** The ECD spectra were simulated by overlapping Gaussian functions for each transition according to:

$$\Delta\varepsilon(E) = \frac{1}{2.297\times10^{-39}} \times \frac{1}{\sqrt{2\pi\sigma}} \sum_{i}^{A} \Delta E_i R_i e^{-[(E-E_i)/(2\sigma)]^2}$$

The $\sigma$ represented the width of the band at $1/e$ height, and $\Delta E_i$ and $R_i$ were the excitation energies and rotatory strengths for transition $i$, respectively. $\sigma$ = 0.20 eV and $R^{\text{velocity}}$ had been used in this work.

**MO Analysis.** The orbital information (NBO plot files) was generated by NBO program[39] of Gaussian 09. The dominantly populated conformers were selected to molecular orbital (MO) analysis. The NBO plot files were used to generate corresponding Gaussian-type grid file by Multiwfn 2.4.[40] After that, the isosurface of generated grid date was generated by VMD software.[41]

**Cytotoxicity Assay.** The following human tumor cell lines were used: HL-60, MMC-7721, A549, MCF-7, and SW480. All cells were cultured in RPMI-1640 or DMEM medium (Hyclone, Logan, UT), supplemented with 10% fetal bovine serum (Hyclone) at 37 °C in a humidified atmosphere with 5% $CO_2$. Cell viability was assessed by conducting colorimetric measurements of the amount of insoluble formazan formed in living cells based on the reduction of 3-(4,5-dimethylthiazol-2-yl)-5-(3-carboxymethoxyphenyl)-2-(4-sulfopheny)-2H-tetrazolium (MTS) (Promega).[36] Briefly, 100 $\mu$L of adherent cells was seeded into each well of a 96-well cell culture plate and allowed to adhere for 12 h before drug addition, while suspended cells were seeded just before drug addition, both with an initial density of 5 × $10^3$–1 × $10^4$ cells/mL in 100 $\mu$L of medium. Each cell line was exposed to the test compound at various concentrations in triplicate for 48 h, with cisplatin (Sigma) and taxol (Sigma) as positive controls. After the incubation, the medium containing compounds was removed , and the cells were incubated for 1–4 h at 37 °C in the dark with 20 $\mu$L of the MTS reagent diluted in 100 $\mu$L of culture medium. The optical density was measured at 490 nm in a 96-well microtiter plate reader (Bio-Rad 680). The $IC_{50}$ value of each compound was calculated by Reed and Muench's method.[42]

**Acetylcholinesterase Inhibitory Activity Assay.** Acetylcholinesterase (AChE) inhibitory activity of the compounds isolated was assayed by the spectrophotometric method developed by Ellman[37] with slightly modification. *S*-acetylthiocholine iodide, 5,5'-dithio-bis-(2-nitrobenzoic) acid (DTNB, Ellman's reagent), and acetylcholinesterase derived from human erythrocytes were purchased from Sigma Chemical. Compounds were dissolved in DMSO. The reaction mixture (totally 200 $\mu$L) containing phosphate buffer (pH 8.0), test compound (50 $\mu$M), and acetyl cholinesterase (0.02 U/mL) was incubated for 20 min (30 °C). Then, the reaction was initiated by the addition of 40 $\mu$L of solution containing DTNB (0.625 mM) and acetylthiocholine iodide (0.625 mM) for AChE inhibitory activity assay. The hydrolysis of acetylthiocholine was monitored at 405 nm every 3 minutes for one hour. Tacrine was used as positive control with final concentration of 0.333 $\mu$M. All the reactions were performed in triplicate. The percentage inhibition was calculated as follows: % inhibition = (E – S)/E × 100 (E is the activity of the enzyme without test compound and S is the activity of enzyme with test compound).

## Electronic Supplementary Material

Supplementary material is available in the online version of this article at http://dx.doi.org/ 10.1007/s13659-012-0082-4 and is accessible for authorized users.

## Acknowledgments

This project was supported financially by the grants from the Chinese Academy of Sciences (KSCX2-EW-Q-10 and KSCX1-YW-R-24), the NSFC (No. 20802082 and 30830115), the Major State Basic Research Development Program of China (No. 2009CB522303 and 2009CB940900), and the project of recruited top talent of sciences and technology of Yunnan Province (2006PY01-47).

**Open Access** This article is distributed under the terms of the Creative Commons Attribution License which permits any use, distribution, and reproduction in any medium, provided the original author(s) and source are credited.

## Electronic supplementary material

Supplementary material, approximately 3.01 MB.
