# Supplementary material for: Four new indole alkaloids from Plantago asiatica
Source: Nat Prod Bioprospect. 2012 Dec 20;2(6):249–54. doi: 10.1007/s13659-012-0082-4 (PMC4131610; doi:10.1007/s13659-012-0082-4)

Electronic Supplementary Material

Four new indole alkaloids from *Plantago asiatica*

Zhong-Hua GAO,[a,b] Ling-Mei KONG,[a] Xi-Sheng ZOU,[a] Yi-Ming SHI,[a,b] Shan-Zhai SHANG,[a] Huai-Rong LUO,[a] Cheng-Qin LIANG,[a] Xiao-Nian LI,[a] Yan LI,[a] Xue DU,[a] Wei-Lie XIAO,[a,]* and Han-Dong SUN[a,]*

[a]State Key Laboratory of Phytochemistry and Plant Resources in West China, Kunming Institute of Botany, Chinese Academy of Sciences, Kunming 650201, Yunnan, China

[b]University of Chinese Academy of Sciences, Beijing 100049, China

Received 17 October 2012; Accepted 12 December 2012

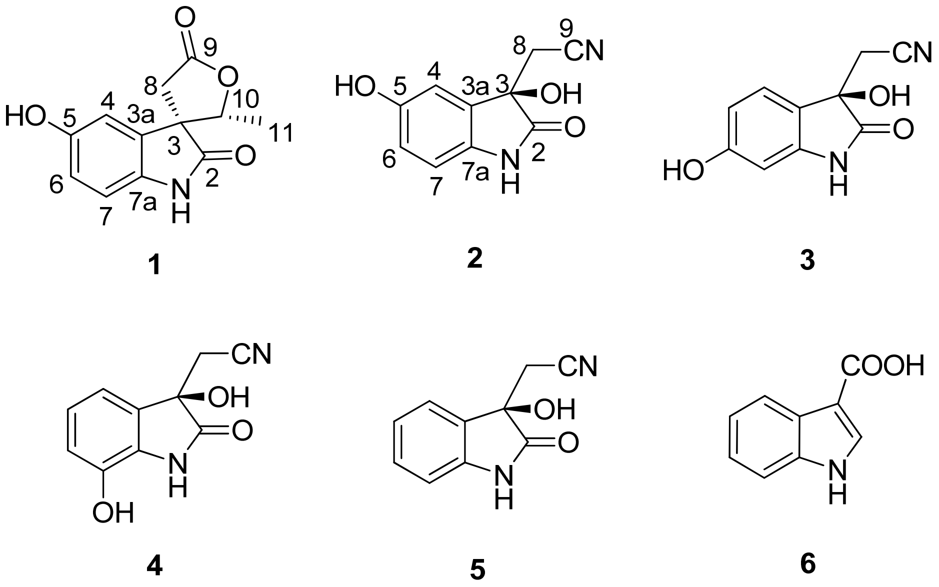

Structures of compounds **1**–**6**

*To whom correspondence should be addressed. E-mail: xwl@mail.kib.ac.cn (W.L. Xiao); hdsun@mail.kib.ac.cn (H.D. Sun)

Contents of Electronic Supplementary Material

No.	Contents	Pages
1	Figure 1S-7S. NMR and MS spectra of compound **1**	3–6
2	Figure 8S. Optimized geometries of configurations of compound **1** at the B3LYP/6-31G(d) level in the gas phase.	7
3	Table 1S. Important thermodynamic parameters (a.u.) of the optimized compound **1** at B3LYP/6-31G(d) level in the gas phase	8
4	Table 2S. Conformational analysis of compound **1**	9
5	Table 3S. Key transitions, oscillator Strengths, and rotatory strengths in the ECD spectra of conformers **1a** and **1b** at B3LYP-SCRF/6-31+G(d,p)//B3LYP/6-31G(d) level with PCM model in MeOH	10-11
6	Table 4S. Optimized Z-Matrixes of compound **1** in the Gas Phase(Å) at B3LYP/6-31G(d) level	12
7	Figure 9S-15S. NMR and MS spectra of compound **2**	13-16
8	Figure 16S. Optimized geometries of configurations of compound **2** at the B3LYP/6-31G(d) level in the gas phase.	17
9	Table 5S. Important thermodynamic parameters (a.u.) of the optimized compound **2** at B3LYP/6-31G(d) level in the gas phase	18
10	Table 6S. Conformational analysis of compound **2**	19
11	Table 7S. Key transitions, oscillator Strengths, and rotatory strengths in the ECD spectra of conformers **2a**, **2b**, **2c**, and **2e** at B3LYP-SCRF/6-31+G(d,p)//B3LYP/6-31G(d) level with PCM model in MeOH	20-22
12	Table 8S. Optimized Z-Matrixes of compound **2** in the Gas Phase(Å) at B3LYP/6-31G(d) level	23-24
13	Figure 17S-24S. NMR, MS and CD spectra of compound **3**	25-29
14	Figure 25S-32S. NMR, MS and CD spectra of compound **4**	30-34

Figure 1S. ^{1}H NMR (400 MHz) spectrum of compound **1** in acetone-d_6.

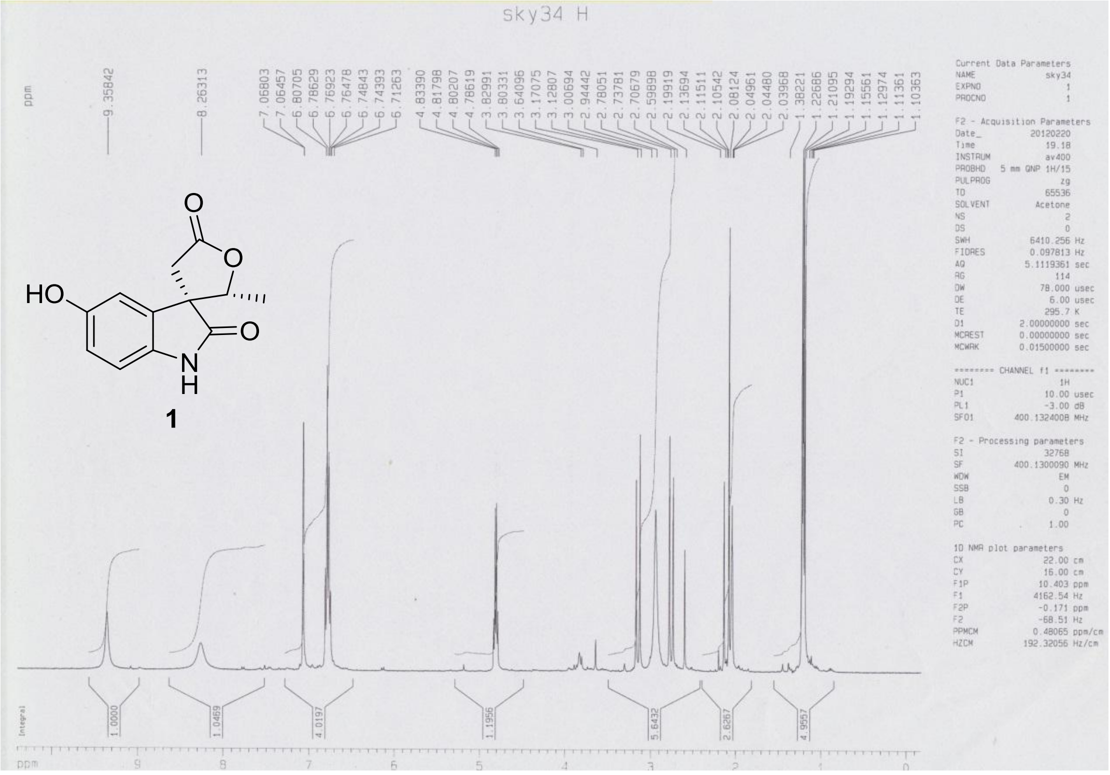

Figure 2S. ^{13}C NMR (100 MHz) spectrum of compound **1** in acetone-d_6.

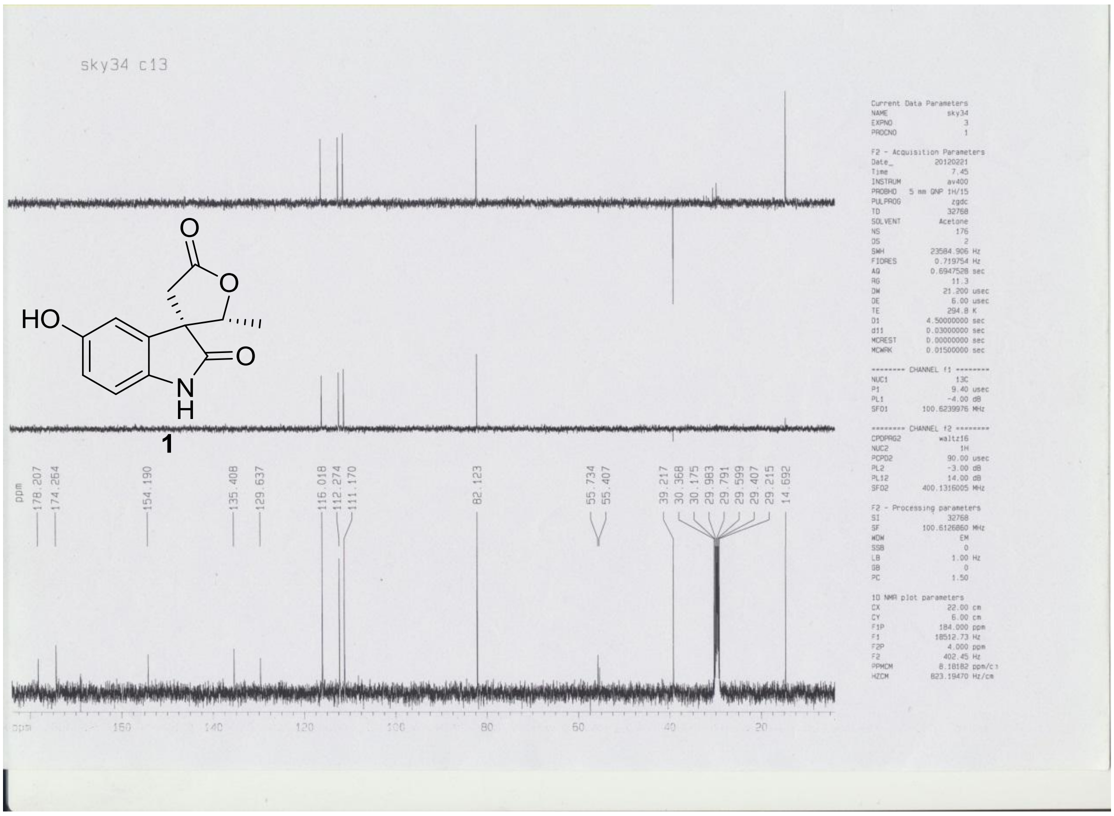

Figure 3S. HSQC (400 MHz) spectrum of compound **1** in acetone-d_6.

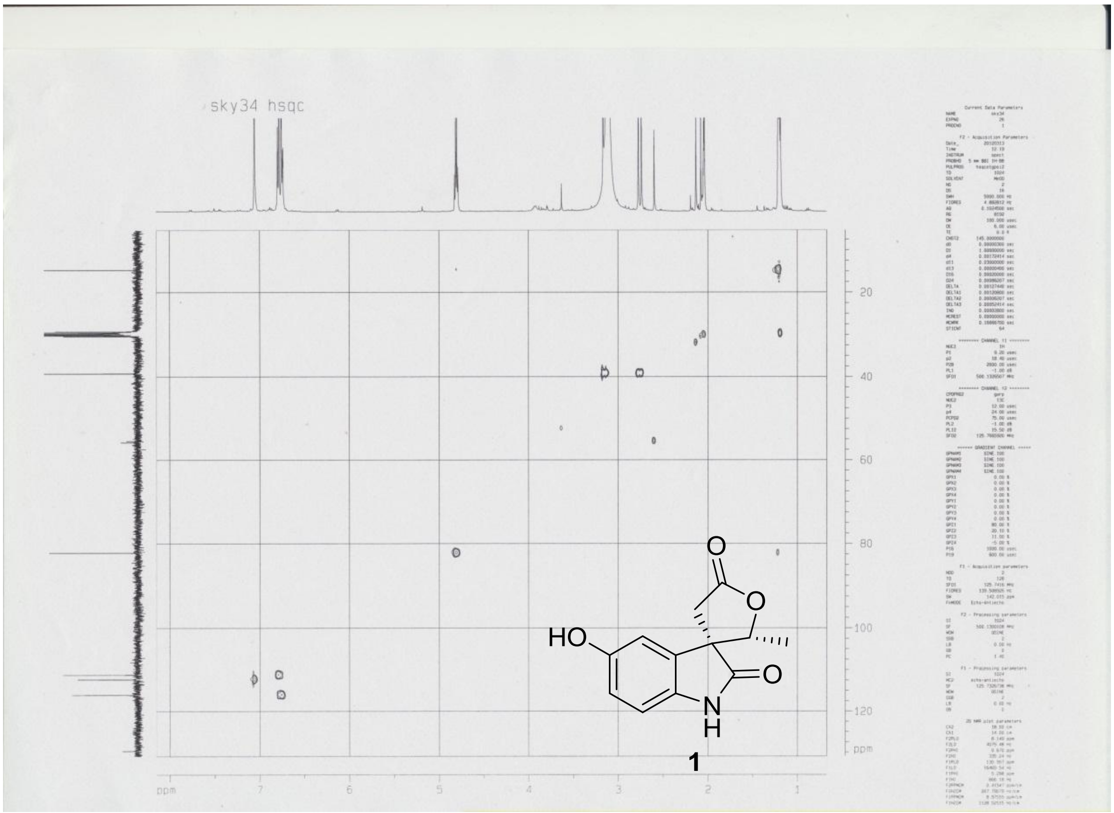

Figure 4S. HMBC (400 MHz) spectrum of compound **1** in acetone-d_6.

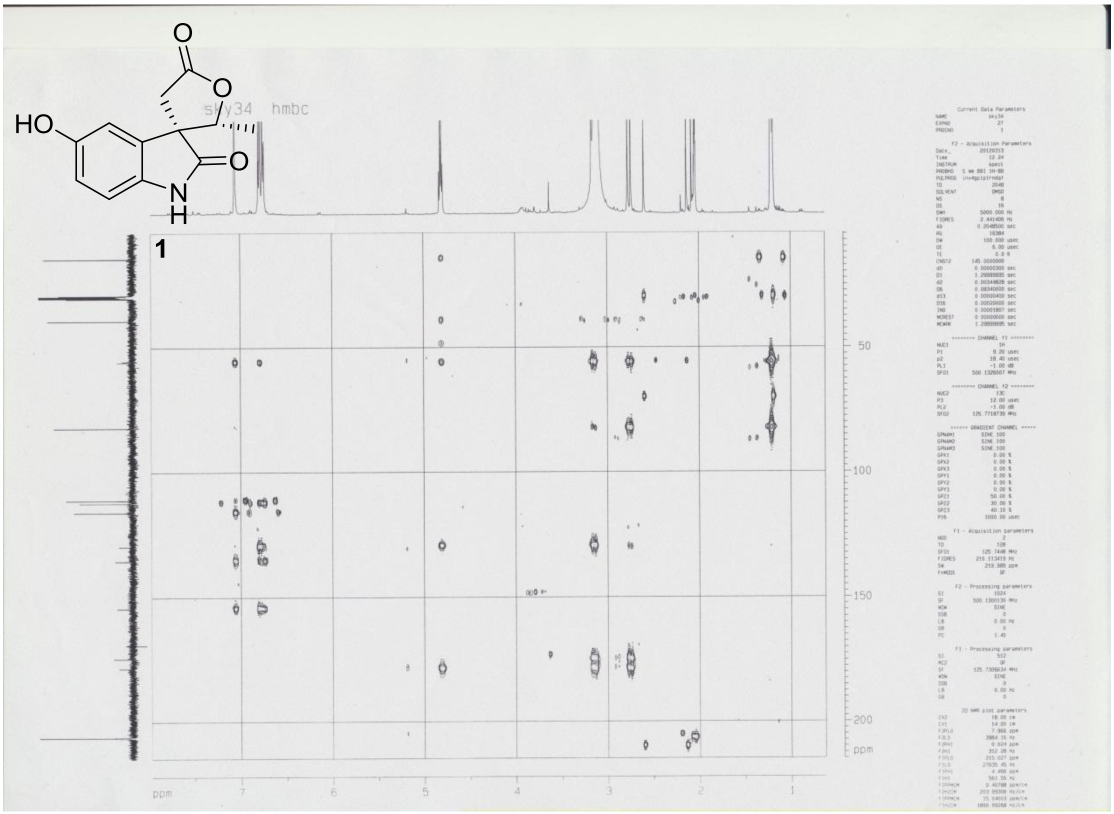

Figure 5S. ^{1}H-^{1}H COSY (400 MHz) spectrum of compound **1** in acetone-d_6.

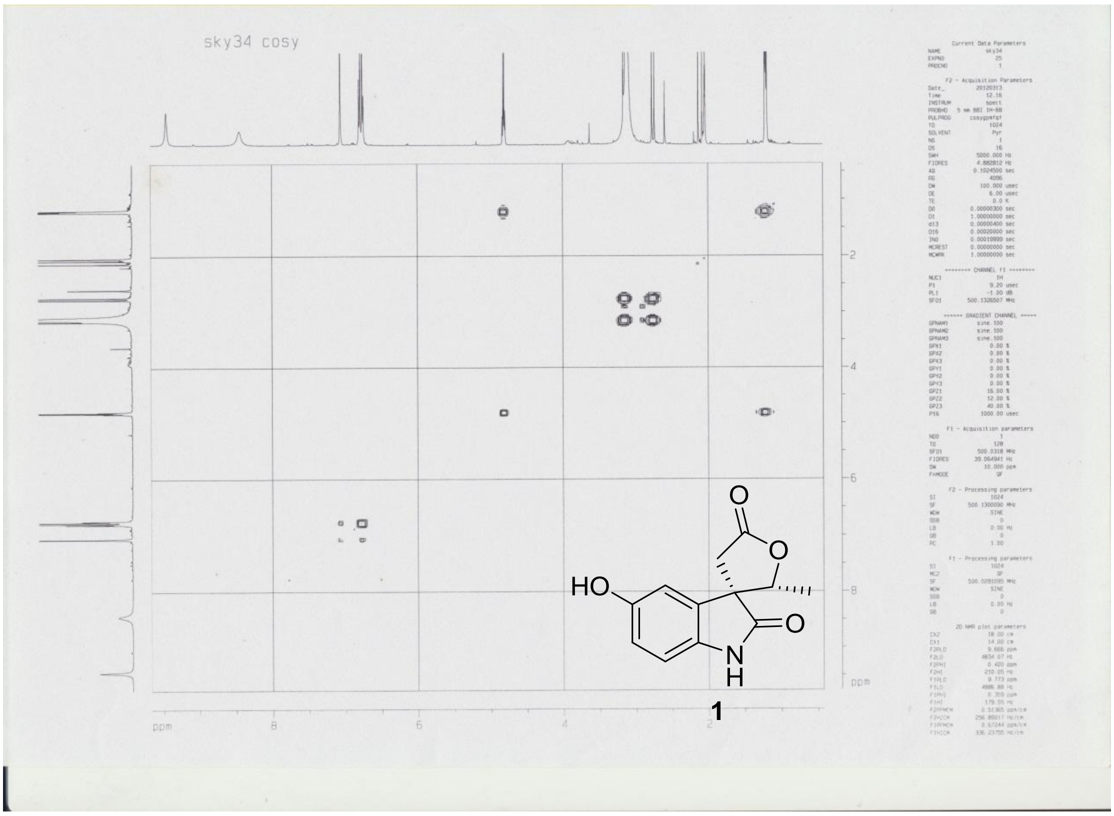

Figure 6S. ROESY (400 MHz) spectrum of compound **1** in acetone-d_6.

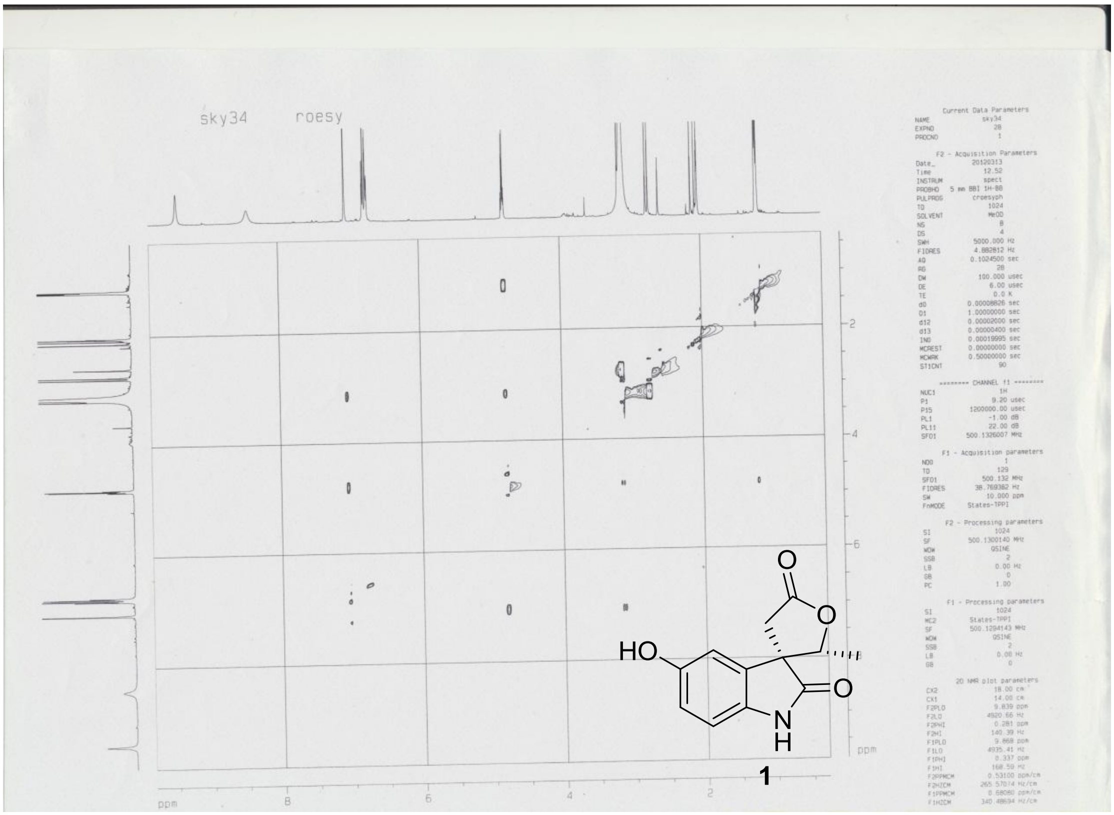

Figure 7S. HREIMS spectrum of compound **1**.

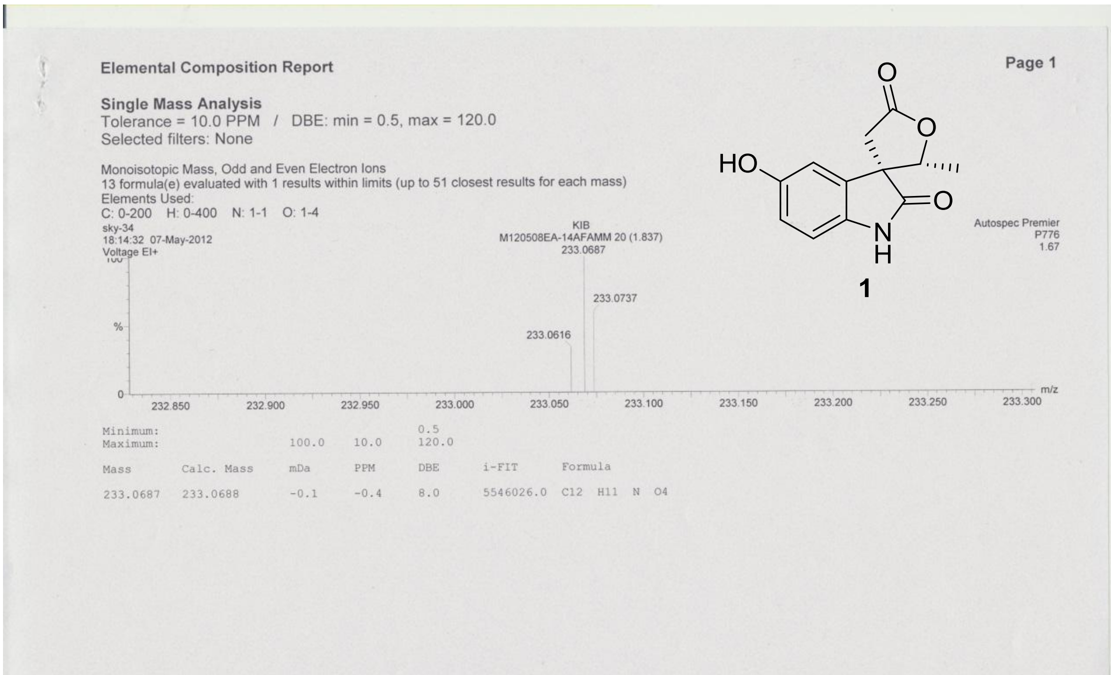

Mass	Calc. Mass	mDa	PPM	DBE	i-FIT	Formula
Minimum:				0.5		
Maximum:		100.0	10.0	120.0		
233.0687	233.0688	-0.1	-0.4	8.0	5546026.0	C12 H11 N O4

Figure 8S. Optimized geometries of configurations of compound **1** at the B3LYP/6-31G(d) level in the gas phase.

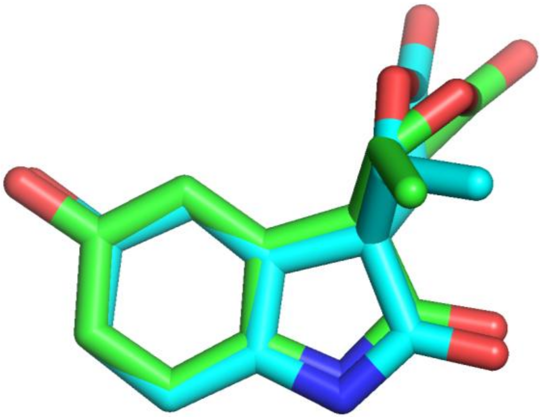

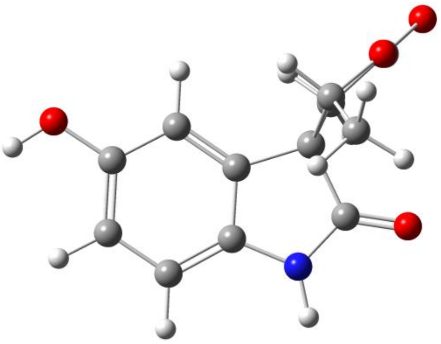

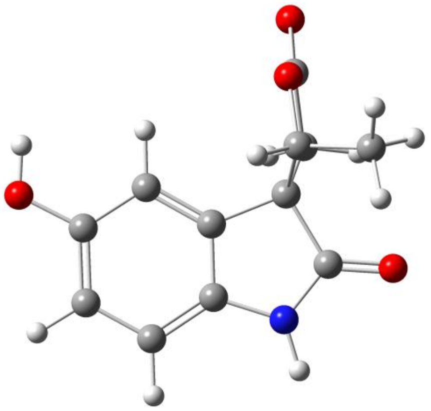

1a **1b**

Table 1S. Important thermodynamic parameters (a.u.) of the optimized compound **1** at B3LYP/6-31G(d) level in the gas phase

Species	*E*	*E'=E+ZPE*	*H*	*G*
**1a**	-819.341191	-819.355304	-819.340247	-819.396225
**1b**	-819.341765	-819.355866	-819.340821	-819.396712

E, E′, H, G: total energy, total energy with zero point energy (ZPE), enthalpy and gibbs free energy in the gas phase at B3LYP/6-31G(d) level.

Table 2S. Conformational analysis of compound **1**

Species	ΔE^a	$P_E\%^b$	$\Delta E'^c$	$P_{E'}\%^d$	ΔG^e	$P_G\%^f$
**1a**	0.36	35.3	0.35	35.5	0.31	37.4
**1b**	0.00	64.7	0.00	64.5	0.00	62.6

[a,c,e] Relative energy, relative energy with ZPE, and relative Gibbs free energy at B3LYP/6-31G(d) level in the gas phase, respectively (kcal/mol). [b,d,f] Conformational distribution calculated by using the respective parameters above at B3LYP/6-31G(d) level in the gas phase.

Table 3S. Key transitions, oscillator Strengths, and rotatory strengths in the ECD spectra of conformers **1a** and **1b** at B3LYP-SCRF/6-31+G(d,p)//B3LYP/6-31G(d) level with PCM model in MeOH

Species	ExitedState	$\Delta E^{a}(eV)$	$\lambda^{b}(nm)$	f^{c}	R_{vel}^{d}	R_{len}^{e}
**1a**	61->62	4.1321	300.05	0.0365	-4.3504	-3.9653
	61->63	4.7549	260.75	0.1846	3.6110	3.6493
	61->64	4.9478	250.58	0.0344	3.0217	2.3406
	61->65	5.1455	240.96	0.0004	0.1037	-0.2170
	59->62	5.3034	233.78	0.0009	-10.3058	-9.0483
	61->66	5.4104	229.16	0.0012	2.2325	1.8357
	60->62	5.6117	220.94	0.1671	-14.8475	-13.4153
	61->68	5.6762	218.43	0.0000	-0.0410	-0.4055
	61->67	5.7469	215.74	0.0013	18.0949	15.3669
	61->67	5.8085	213.45	0.1232	-24.1515	-24.5094
	61->69	5.9219	209.36	0.0246	1.8312	1.2467
	61->70	6.0444	205.12	0.0210	-18.5791	-17.4601
	61->71	6.1303	202.25	0.0026	-0.4200	-1.4708
	59->63	6.1740	200.82	0.0028	6.0956	6.4604
	60->64	6.2425	198.61	0.0683	0.6202	-0.3682
	61->72	6.3031	196.70	0.0011	-0.9240	-0.7895
	57->62	6.3823	194.26	0.1908	26.1994	28.7934
	59->64	6.4552	192.07	0.0106	-16.0692	-16.7100
	60->65	6.5361	189.69	0.0155	-36.4756	-36.1559
	57->62	6.5677	188.78	0.0839	14.9696	14.3170
	59->64	6.6734	185.79	0.0117	6.9511	6.7358
	61->73	6.7068	184.86	0.0044	-0.4608	-0.5757
**1b**	61->62	4.1155	301.26	0.0361	-4.1633	-3.8873
	61->63	4.7459	261.24	0.1720	3.6114	3.3143
	61->64	4.9272	251.63	0.0408	2.0323	1.9047
	61->65	5.1425	241.10	0.0004	0.3574	-0.0832
	59->62	5.2898	234.38	0.0009	-9.4000	-8.1666
	61->66	5.3944	229.84	0.0007	1.7329	1.4263
	60->62	5.6040	221.24	0.1636	-6.7961	-6.1907
	61->68	5.7218	216.69	0.0008	10.1599	9.1542
	61->68	5.7458	215.78	0.0068	-2.0270	-3.4750
	61->67	5.8048	213.59	0.1160	-23.6494	-24.2818
	61->69	5.9249	209.26	0.0296	-0.9770	-1.4229
	61->70	6.0041	206.50	0.0123	-12.5159	-12.3099
	61->71	6.1719	200.88	0.0041	-4.3142	-4.9243
	59->63	6.1796	200.63	0.0042	11.5165	12.1058
	60->64	6.2455	198.52	0.0824	0.3780	-2.3727
	61->71	6.2876	197.19	0.0020	-2.4339	-1.4494
	57->62	6.3826	194.25	0.1720	25.5787	29.1311

	59->64	6.4485	192.27	0.0088	-12.7220	-13.3221
	60->65	6.5410	189.55	0.0154	-34.1982	-34.3220
	57->62	6.5667	188.81	0.0992	5.0221	5.0445
	59->64	6.6660	186.00	0.0081	0.8552	0.5263
	61->73	6.7127	184.70	0.0033	0.6308	0.2323

[a]Excitation energy. [b]Wavelength. [c]Oscillator strength. [d]Rotatory strength in velocity form (10^{-40} cgs.). [e]Rotatory strength in length form (10^{-40} cgs.).

Table 4S. Optimized Z-Matrixes of compound **1** in the Gas Phase(Å) at B3LYP/6-31G(d) level

**1a**				**1b**			
C	2.842825	-0.938293	0.159106	C	2.842952	-0.916329	0.163636
C	3.474375	0.304856	0.062176	C	3.469949	0.330861	0.066513
C	2.727271	1.481027	-0.089596	C	2.717946	1.499128	-0.085054
C	1.346451	1.373593	-0.145655	C	1.334185	1.387561	-0.141983
C	0.702367	0.128580	-0.054624	C	0.695798	0.144086	-0.051237
C	1.441462	-1.032615	0.104227	C	1.443526	-1.015813	0.109009
N	0.376510	2.383036	-0.286791	N	0.360895	2.395288	-0.282774
C	-0.904435	1.878497	-0.339702	C	-0.918318	1.887637	-0.331586
C	-0.796867	0.347547	-0.129948	C	-0.803712	0.356116	-0.127746
C	-1.542585	-0.195642	1.143619	C	-1.547601	-0.199644	1.138781
O	-1.920207	-1.547141	0.780593	O	-1.874238	-1.566317	0.775130
C	-1.977466	-1.711836	-0.572659	C	-1.925141	-1.727895	-0.579244
C	-1.513187	-0.429230	-1.248755	C	-1.510963	-0.424984	-1.249193
O	-1.918682	2.520076	-0.545415	O	-1.937200	2.524695	-0.529131
O	-2.341051	-2.735686	-1.086638	O	-2.242281	-2.766204	-1.095840
C	-2.782000	0.565024	1.597237	C	-2.818174	0.521896	1.569118
O	3.533304	-2.110329	0.310485	O	3.650231	-2.011475	0.313227
H	4.559965	0.363806	0.105866	H	4.553211	0.371851	0.112325
H	3.227304	2.442493	-0.160984	H	3.212502	2.463504	-0.156579
H	0.976211	-2.010055	0.186587	H	0.959590	-1.987040	0.191768
H	0.567271	3.359748	-0.462885	H	0.549280	3.373425	-0.453230
H	-0.837352	-0.295518	1.972272	H	-0.852173	-0.272894	1.978420
H	-2.390174	0.129502	-1.597999	H	-2.408315	0.112729	-1.579271
H	-0.883755	-0.654728	-2.111691	H	-0.887001	-0.621054	-2.123162
H	-3.278577	-0.011437	2.383901	H	-3.303635	-0.062167	2.357153
H	-2.510163	1.545278	1.999369	H	-2.586160	1.516265	1.960780
H	-3.486231	0.724917	0.777091	H	-3.518440	0.645830	0.739271
H	4.483758	-1.919651	0.331249	H	3.100097	-2.808781	0.358775

Figure 9S. ^{1}H NMR (400 MHz) spectrum of compound **2** in C_5D_5N.

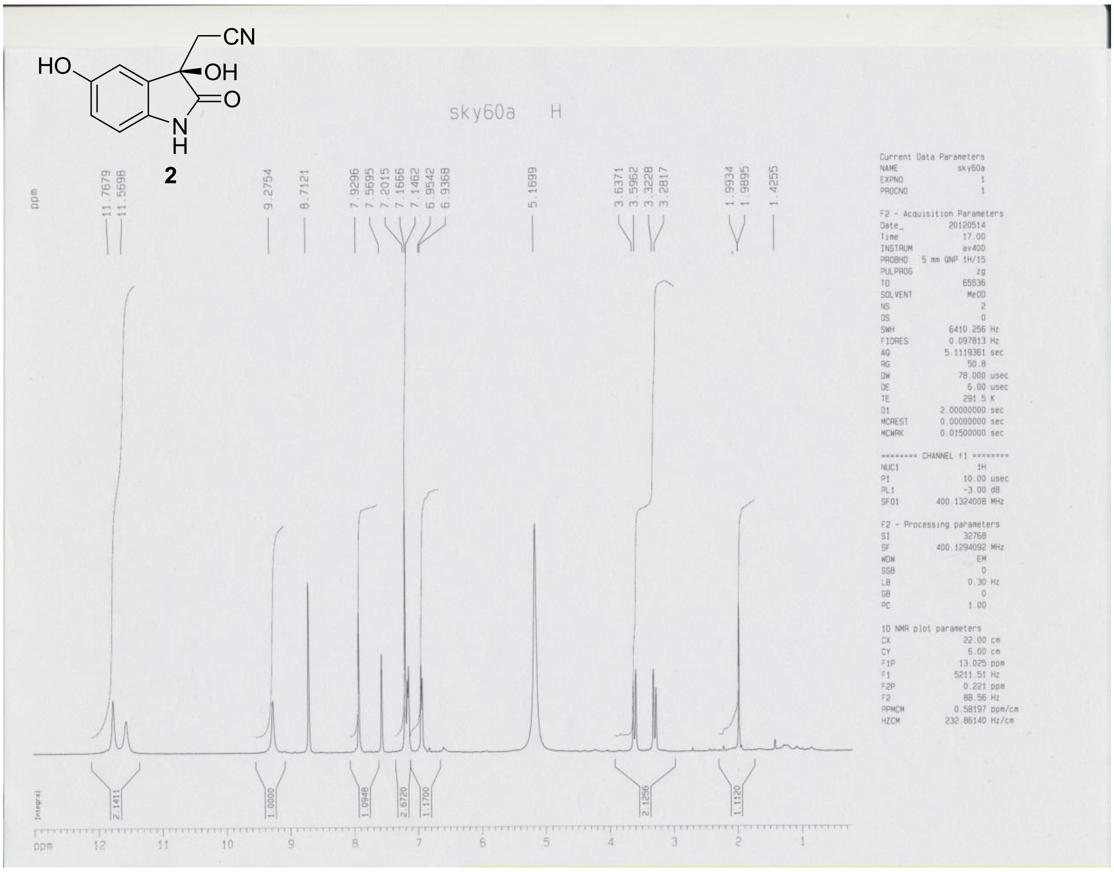

Figure 10S. ^{13}C NMR (100 MHz) spectrum of compound **2** in C_5D_5N.

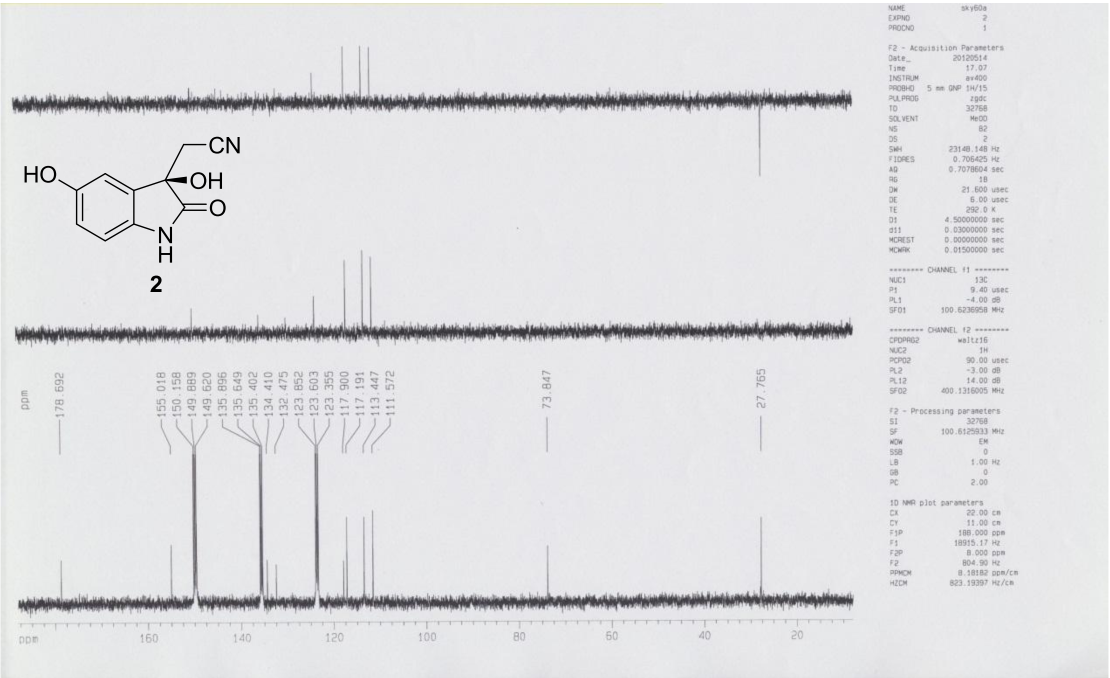

Figure 11S. HSQC (400 MHz) spectrum of compound **2** in C_5D_5N.

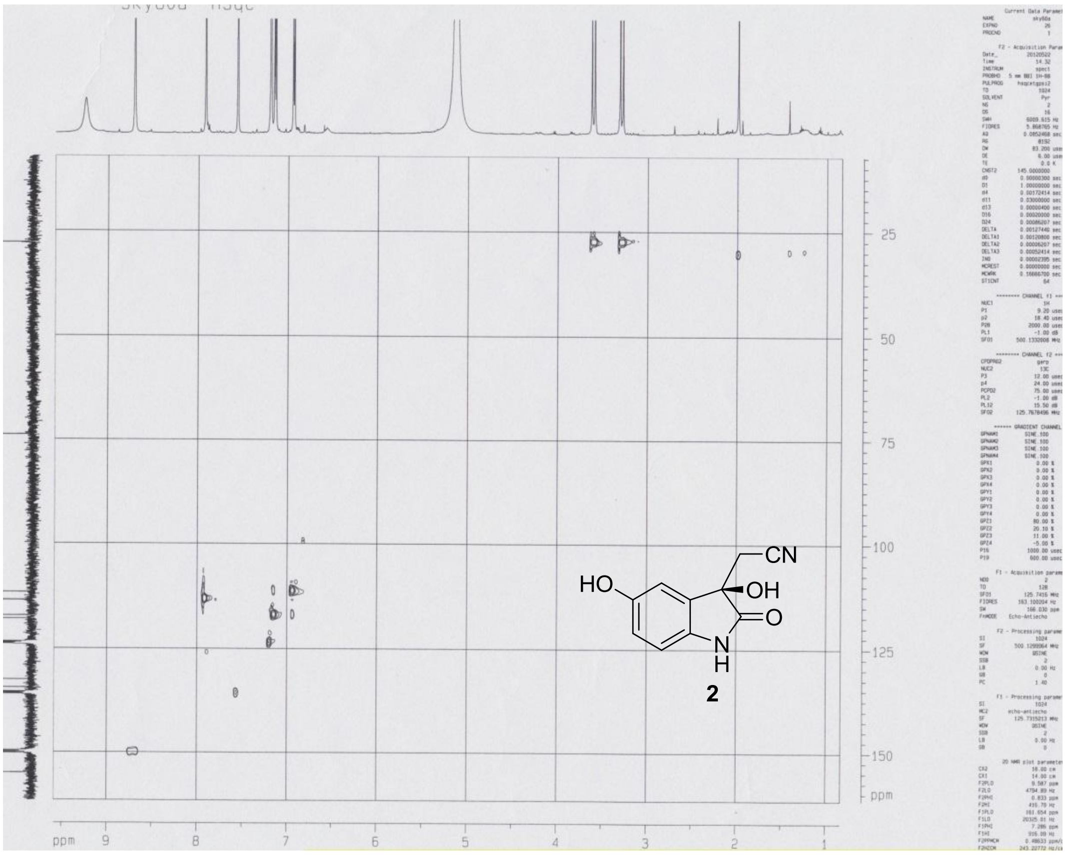

Figure 12S. HMBC (400 MHz) spectrum of compound **2** in C_5D_5N.

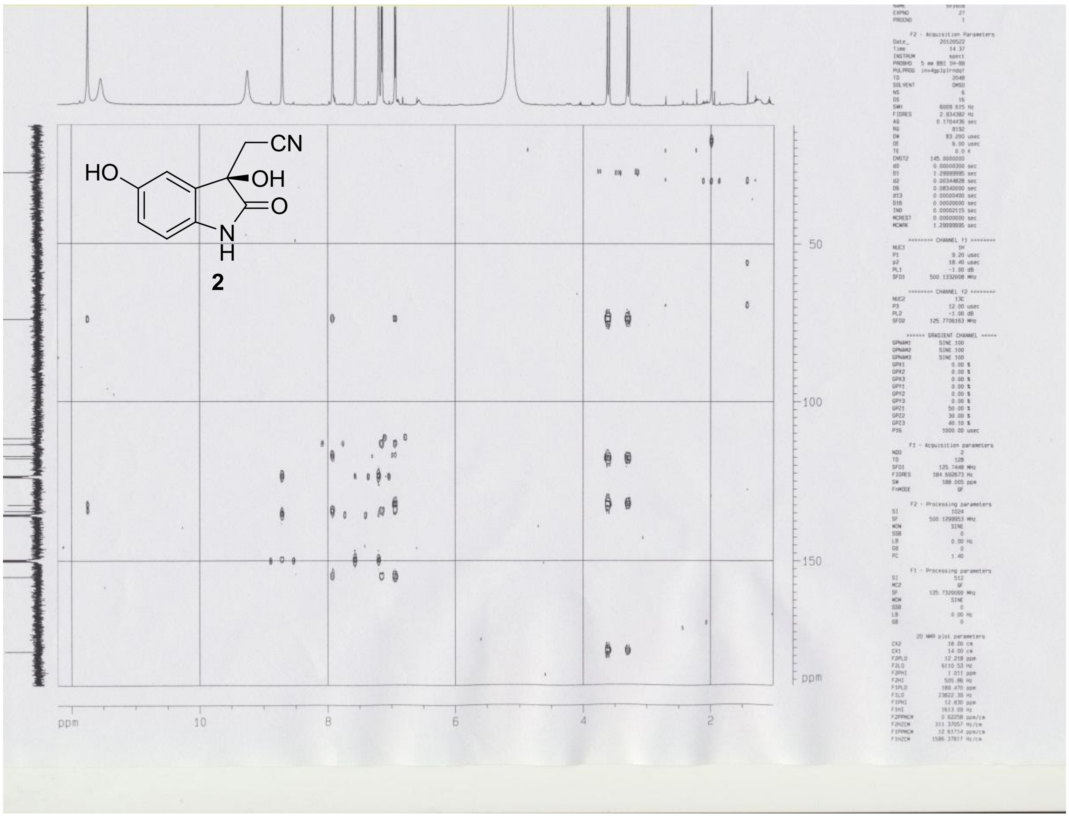

Figure 13S. ^{1}H-^{1}H COSY (400 MHz) spectrum of compound **2** in C_5D_5N.

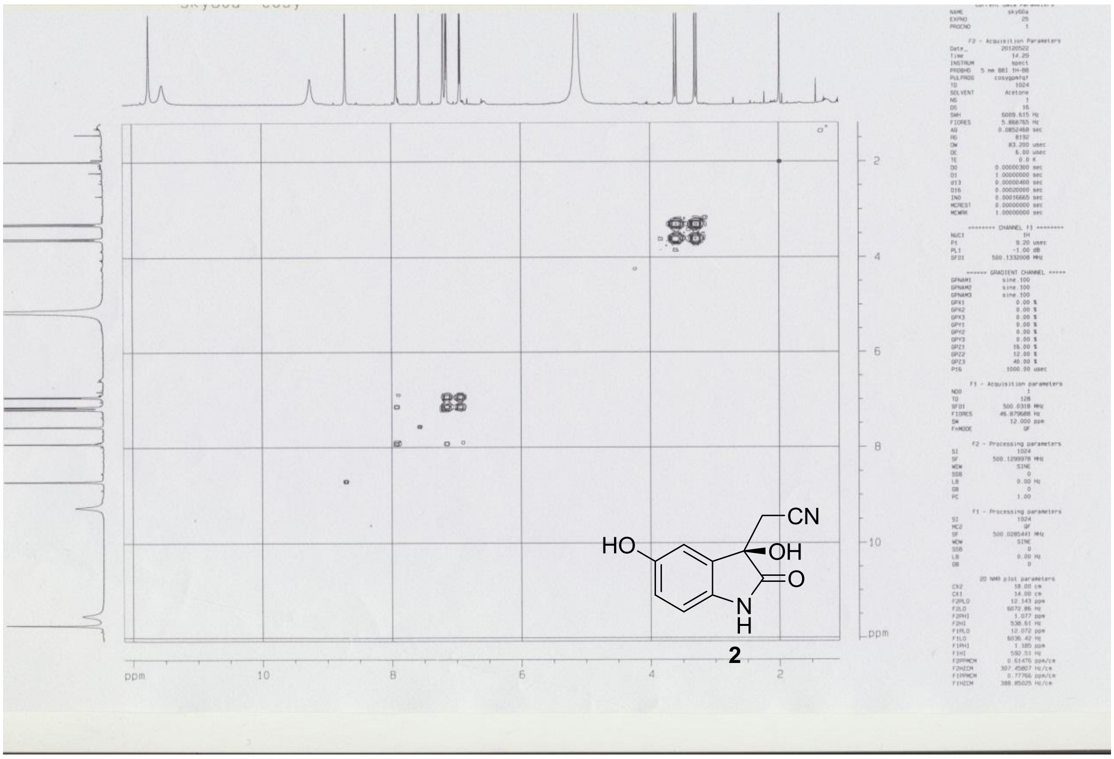

Figure 14S. ROESY (400 MHz) spectrum of compound **2** in C_5D_5N.

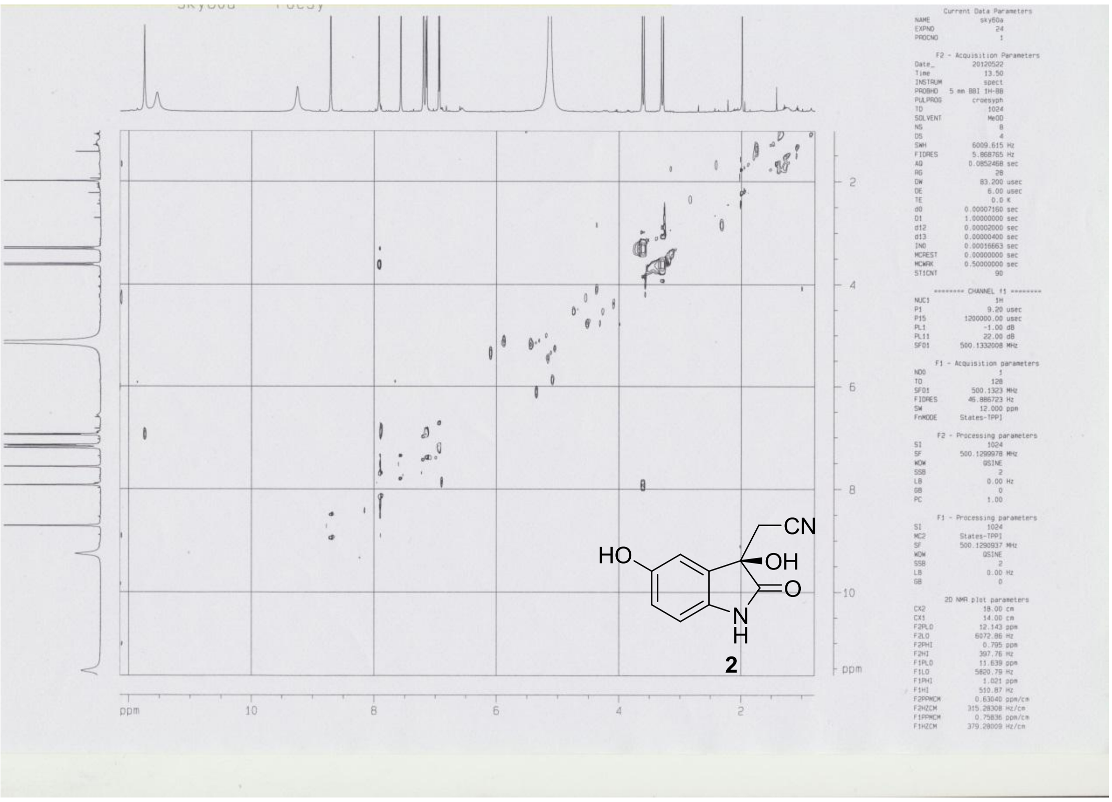

Figure 15S. HREIMS spectrum of compound **2**.

Elemental Composition Report

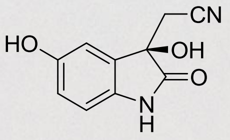

2

Page 1

Single Mass Analysis
Tolerance = 10.0 PPM / DBE: min = -10.0, max = 120.0
Selected filters: None

Monoisotopic Mass, Odd and Even Electron Ions
12 formula(e) evaluated with 1 results within limits (up to 51 closest results for each mass)
Elements Used:
C: 0-200 H: 0-400 N: 2-2 O: 1-3

sky-57a | KIB | Autospec Premier
09:22:52 04-Jun-2012 | M120604EA-01AFAMM 20 (1.838) | P776
Voltage EI+ | 204.0546 | 1.67

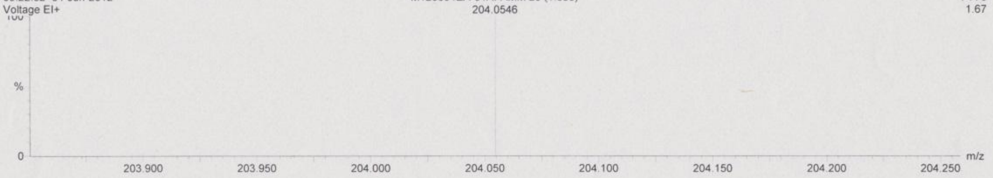

Minimum:				-10.0		
Maximum:		100.0	10.0	120.0		
Mass	Calc. Mass	mDa	PPM	DBE	i-FIT	Formula
204.0546	204.0535	1.1	5.4	8.0	5546026.0	C10 H8 N2 O3

Figure 16S. Optimized geometries of configurations of compound **2** at the B3LYP/6-31G(d) level in the gas phase.

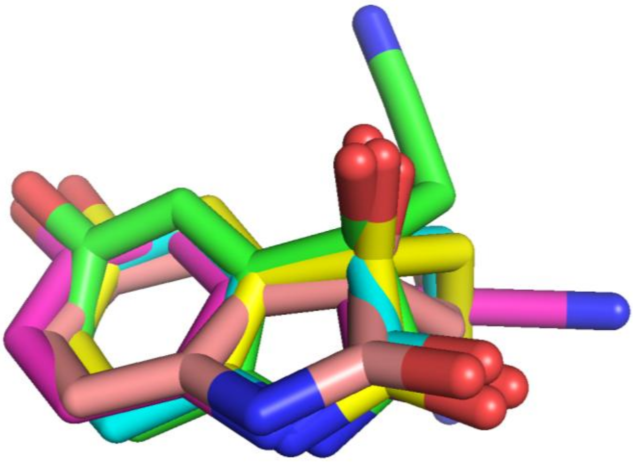

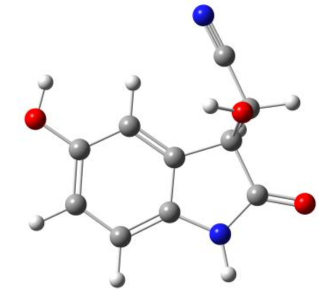

2a

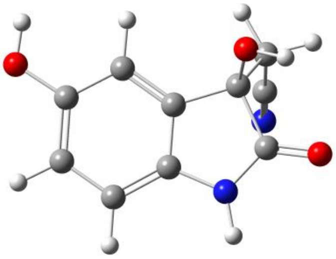

2b

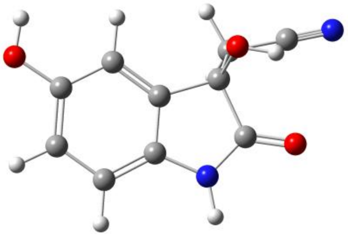

2c

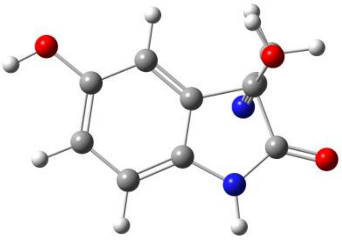

2d

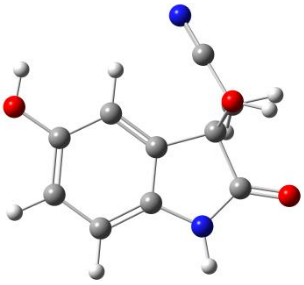

2e

Table 5S. Important thermodynamic parameters (a.u.) of the optimized compound **2** at B3LYP/6-31G(d) level in the gas phase

Species	*E*	*E'=E+ZPE*	*H*	*G*
**2a**	-720.851050	-720.864073	-720.850106	-720.903415
**2b**	-720.852210	-720.865093	-720.851266	-720.904377
**2c**	-720.850876	-720.863825	-720.849932	-720.903198
**2d**	-720.849611	-720.862707	-720.848666	-720.902291
**2e**	-720.853462	-720.866294	-720.852518	-720.905406

E, E′, H, G: total energy, total energy with zero point energy (ZPE), enthalpy and gibbs free energy in the gas phase at B3LYP/6-31G(d) level.

Table 6S. Conformational analysis of compound **2**

Species	ΔE^{a}	$P_E\%^{b}$	$\Delta E'^{c}$	$P_{E'}\%^{d}$	ΔG^{e}	$P_G\%^{f}$
**2a**	1.51	5.5	1.39	6.5	1.25	7.6
**2b**	0.79	18.6	0.75	19.1	0.65	21.1
**2c**	1.62	4.5	1.55	5.0	1.39	6.1
**2d**	2.42	1.2	2.25	1.5	1.95	2.3
**2e**	0.00	70.2	0.00	68.0	0.00	62.9

[a,c,e] Relative energy, relative energy with ZPE, and relative Gibbs free energy at B3LYP/6-31G(d) level in the gas phase, respectively (kcal/mol). [b,d,f] Conformational distribution calculated by using the respective parameters above at B3LYP/6-31G(d) level in the gas phase.

Table 7S. Key transitions, oscillator Strengths, and rotatory strengths in the ECD spectra of conformers **2a**, **2b**, **2c**, and **2e** at B3LYP-SCRF/6-31+G(d,p)//B3LYP/6-31G(d) level with PCM model in MeOH

Species	ExitedState	$\Delta E^a(eV)$	$\lambda^b(nm)$	f^c	R_{vel}^d	R_{len}^e
**2a**	53->54	3.9262	315.79	0.0460	2.7457	2.1400
	53->55	4.7006	263.76	0.1002	28.7807	28.1814
	51->54	4.9110	252.46	0.0365	-75.0941	-74.0734
	53->56	5.1298	241.69	0.0146	-1.1008	-1.7060
	53->57	5.3107	233.46	0.0047	-1.2154	1.3956
	52->54	5.5650	222.79	0.1919	61.3622	64.5518
	53->58	5.6654	218.85	0.1737	46.7247	42.8010
	53->59	5.7487	215.68	0.0027	0.2551	0.5239
	53->60	5.7874	214.23	0.0016	-0.1707	-0.1464
	52->55	5.9602	208.02	0.0063	-0.2820	-0.9914
	53->61	6.0248	205.79	0.0082	12.6935	12.3065
	53->62	6.0920	203.52	0.0202	19.2127	19.7509
	53->63	6.1509	201.57	0.0599	-16.0714	-14.9753
	50->54	6.2345	198.87	0.0387	-32.3681	-33.2138
	53->64	6.4193	193.14	0.0078	10.2558	10.1020
	52->56	6.4662	191.74	0.0111	-19.4528	-22.3554
	49->54	6.4973	190.82	0.0257	-7.6305	-8.5182
	53->65	6.5687	188.75	0.0019	-7.6441	-5.9059
	52->57	6.6124	187.50	0.1131	-64.5632	-69.1751
	52->57	6.7098	184.78	0.1905	70.3223	71.0305
	51->58	6.7606	183.39	0.1009	9.0037	10.6612
	52->58	6.8821	180.16	0.0246	14.2903	14.7461
**2b**	53->54	4.0844	303.56	0.0465	6.5853	7.0826
	53->55	4.7539	260.81	0.1536	-5.5624	-5.5718
	53->56	5.1795	239.38	0.0001	0.6697	0.3873
	51->54	5.2108	237.94	0.0053	-24.9305	-25.4960
	53->57	5.4005	229.58	0.0001	0.1403	0.2139
	53->59	5.5138	224.86	0.0020	4.8631	5.8885
	53->58	5.5610	222.95	0.0249	1.0322	0.4816
	52->54	5.7584	215.31	0.3109	6.0264	5.6601
	53->60	5.8239	212.89	0.0321	1.0907	1.4222
	53->61	5.9465	208.50	0.0061	0.4997	0.0449
	53->63	6.1256	202.40	0.0029	5.9028	6.2190
	51->55	6.1668	201.05	0.0074	-14.9330	-15.7201
	53->62	6.1774	200.71	0.0498	20.8746	21.3152
	49->54	6.3191	196.20	0.0458	32.8915	33.3151
	50->54	6.3714	194.59	0.0782	-21.9998	-20.6566
	53->64	6.4464	192.33	0.0049	-0.3425	-0.6507
	52->56	6.5950	188.00	0.0181	4.8830	2.3720

	53->65	6.6225	187.22	0.0183	-5.5223	-4.3531
	52->55	6.6852	185.46	0.2604	-31.4123	-32.6476
	51->56	6.8066	182.15	0.0075	10.0642	8.7058
	52->57	6.8342	181.42	0.0060	-30.0579	-29.1438
	52->58	6.9156	179.28	0.1890	-13.4076	-14.3593
**2c**	53->54	4.0407	306.84	0.0414	7.2627	7.2751
	53->55	4.7500	261.02	0.1563	4.0077	3.9997
	51->54	5.1406	241.18	0.0211	-56.6370	-57.3033
	53->56	5.1554	240.49	0.0062	-1.4331	-1.9173
	53->57	5.4487	227.55	0.0022	-2.2191	-1.3508
	52->54	5.6493	219.47	0.1167	17.0797	17.4853
	53->59	5.6856	218.07	0.0236	-4.0991	-4.4259
	52->54	5.7417	215.94	0.1693	33.6725	34.417
	53->61	5.8447	212.13	0.0200	2.5721	2.7532
	53->60	5.9082	209.85	0.0970	3.0495	3.4401
	51->55	6.0759	204.06	0.0104	18.7200	18.7525
	53->62	6.1012	203.21	0.0258	29.9517	29.8096
	53->63	6.2866	197.22	0.0764	5.1474	4.0657
	50->54	6.3235	196.07	0.0559	-2.1478	-0.9341
	49->54	6.3825	194.26	0.0107	-3.5254	-3.2432
	53->65	6.4487	192.26	0.0013	5.8717	6.3446
	53->64	6.5354	189.71	0.0148	-10.4245	-13.0818
	53->64	6.5499	189.29	0.0406	16.0386	17.8565
	52->56	6.6619	186.11	0.2360	-75.1210	-76.7719
	51->56	6.7563	183.51	0.0130	-2.7400	-3.8824
	53->66	6.9390	178.68	0.0053	1.5335	1.8404
	52->57	6.9492	178.41	0.0039	3.1310	2.9902
**2e**	53->54	4.0487	306.23	0.0454	4.8393	4.8905
	53->55	4.7629	260.32	0.1471	0.0377	-0.0035
	53->56	5.1727	239.69	0.0089	9.9273	9.3445
	51->54	5.1854	239.1	0.0097	-31.5975	-31.372
	53->57	5.3537	231.59	0.0051	0.2113	2.4317
	53->58	5.6212	220.56	0.0964	20.6466	22.3664
	53->58	5.7088	217.18	0.1845	26.4168	24.3639
	53->59	5.7630	215.14	0.0672	15.4001	14.6339
	53->60	5.7971	213.87	0.0057	1.1485	1.2996
	53->61	5.9961	206.77	0.0088	-5.8446	-5.9757
	53->62	6.1249	202.43	0.0030	0.1258	0.2438
	51->55	6.1634	201.16	0.0040	5.7880	5.3115
	50->54	6.3267	195.97	0.1004	20.3579	19.5957
	53->63	6.3378	195.63	0.0141	5.8555	6.1264
	51->55	6.3739	194.52	0.0146	-25.1883	-24.3638
	53->64	6.4380	192.58	0.0016	-3.0487	-3.4843

	52->56	6.5881	188.19	0.0315	-22.2041	-26.3487
	53->65	6.6102	187.56	0.0121	-4.2284	-1.7229
	52->55	6.6794	185.62	0.1962	-71.3673	-73.7548
	51->56	6.7743	183.02	0.0873	6.1376	5.8723
	52->57	6.8134	181.97	0.0899	39.3570	41.0805
	53->66	6.9494	178.41	0.0019	-0.0601	0.1268

[a]Excitation energy. [b]Wavelength. [c]Oscillator strength. [d]Rotatory strength in velocity form (10^{-40} cgs.). [e]Rotatory strength in length form (10^{-40} cgs.).

Table 8S. Optimized Z-Matrixes of compound **2** in the Gas Phase(Å) at B3LYP/6-31G(d) level

**2a**				**2b**			
C	-2.590652	0.343182	0.099631	C	-2.682690	-0.266813	-0.218261
C	-2.844081	-0.993899	-0.229280	C	-2.748822	0.884451	0.574909
C	-1.797620	-1.908731	-0.379067	C	-1.595244	1.607435	0.890053
C	-0.497465	-1.454097	-0.196356	C	-0.381416	1.151630	0.393443
C	-0.234447	-0.116505	0.125462	C	-0.304337	-0.003608	-0.393358
C	-1.273751	0.790923	0.286496	C	-1.448277	-0.722016	-0.706776
N	0.716315	-2.168495	-0.275423	N	0.909963	1.707241	0.548097
C	1.809570	-1.378170	0.001025	C	1.852308	0.997665	-0.142515
C	1.262502	0.068156	0.279608	C	1.143886	-0.248130	-0.746783
C	1.924818	1.015124	-0.743976	C	1.741358	-1.549928	-0.139988
O	2.974450	-1.719118	0.016781	O	3.027810	1.265959	-0.321788
O	1.667212	0.548838	1.550682	O	1.362462	-0.269320	-2.146584
O	-3.672338	1.169898	0.230987	O	-3.861818	-0.907810	-0.485988
C	1.463264	2.398556	-0.611664	C	1.538879	-1.699096	1.302312
N	1.071938	3.487303	-0.519049	N	1.379260	-1.805956	2.446889
H	-3.874145	-1.306974	-0.365038	H	-3.718711	1.205237	0.940437
H	-2.007156	-2.944510	-0.629894	H	-1.658709	2.499964	1.505277
H	-1.067384	1.827539	0.541186	H	-1.393883	-1.612343	-1.329903
H	0.801920	-3.156430	-0.471595	H	1.115533	2.587788	1.000393
H	3.007318	0.965418	-0.580357	H	1.295161	-2.402585	-0.663658
H	1.723797	0.667984	-1.763499	H	2.818151	-1.553742	-0.349338
H	1.174472	0.061439	2.230546	H	2.279955	0.035239	-2.279213
H	-3.364007	2.069690	0.421075	H	-3.683568	-1.688733	-1.032222
**2c**				**2d**			
C	2.851690	-0.715427	0.000067	C	-2.678986	-0.318045	-0.166500
C	3.173892	0.606193	-0.329737	C	-2.757123	0.880131	0.550270
C	2.181958	1.586675	-0.422160	C	-1.612049	1.646419	0.808034
C	0.866265	1.214278	-0.176544	C	-0.395426	1.179013	0.335454
C	0.534612	-0.108168	0.141379	C	-0.308199	-0.028782	-0.373885
C	1.517005	-1.082733	0.232586	C	-1.439745	-0.780713	-0.638844
N	-0.303445	2.006454	-0.186455	N	0.883794	1.761967	0.430274
C	-1.406795	1.283629	0.203149	C	1.853501	1.009224	-0.193685
C	-0.964810	-0.207231	0.305305	C	1.138357	-0.269212	-0.757068
C	-1.590696	-1.041151	-0.854568	C	1.785844	-1.526351	-0.134807
O	-2.520428	1.700777	0.453723	O	3.034197	1.271644	-0.293051
O	-1.333137	-0.738592	1.559245	O	1.361350	-0.436191	-2.148612
O	3.885763	-1.608263	0.079580	O	-3.774837	-1.088686	-0.446646
C	-3.039807	-1.212340	-0.719129	C	1.635193	-1.615519	1.317974
N	-4.182210	-1.378216	-0.600709	N	1.519231	-1.686969	2.470295
H	4.214862	0.855687	-0.507068	H	-3.722203	1.227606	0.913419
H	2.445404	2.610191	-0.671920	H	-1.689298	2.576959	1.362369

H	1.261489	-2.106111	0.500149	H	-1.395058	-1.709619	-1.198936
H	-0.316187	3.017102	-0.224362	H	1.098276	2.628774	0.903830
H	-1.363121	-0.583804	-1.824635	H	1.342247	-2.410947	-0.605159
H	-1.126713	-2.034012	-0.842231	H	2.851246	-1.503833	-0.391020
H	-2.252950	-0.454493	1.718952	H	0.960655	0.316524	-2.612685
H	3.539333	-2.475960	0.338794	H	-4.560935	-0.681573	-0.050986
**2e**							
C	-2.597050	0.349312	0.133540				
C	-2.854375	-0.976346	-0.234834				
C	-1.809408	-1.887362	-0.415386				
C	-0.510360	-1.439702	-0.216608				
C	-0.243777	-0.113605	0.146034				
C	-1.278815	0.791804	0.326815				
N	0.705991	-2.155772	-0.325629				
C	1.782509	-1.378279	0.004848				
C	1.253139	0.063014	0.260981				
C	1.866305	0.999772	-0.818006				
O	2.949855	-1.709870	0.118507				
O	1.637392	0.474487	1.558683				
O	-3.676388	1.173678	0.292600				
C	1.461178	2.394500	-0.638088				
N	1.117569	3.494330	-0.498485				
H	-3.885326	-1.283527	-0.376579				
H	-2.020332	-2.914543	-0.698121				
H	-1.071967	1.819769	0.612794				
H	0.781762	-3.155943	-0.454591				
H	2.958930	0.921905	-0.756040				
H	1.572933	0.669227	-1.821854				
H	2.562550	0.187955	1.674151				
H	-3.366420	2.060239	0.534942				

Figure 17S. ^{1}H NMR (500 MHz) spectrum of compound **3** in C_5D_5N.

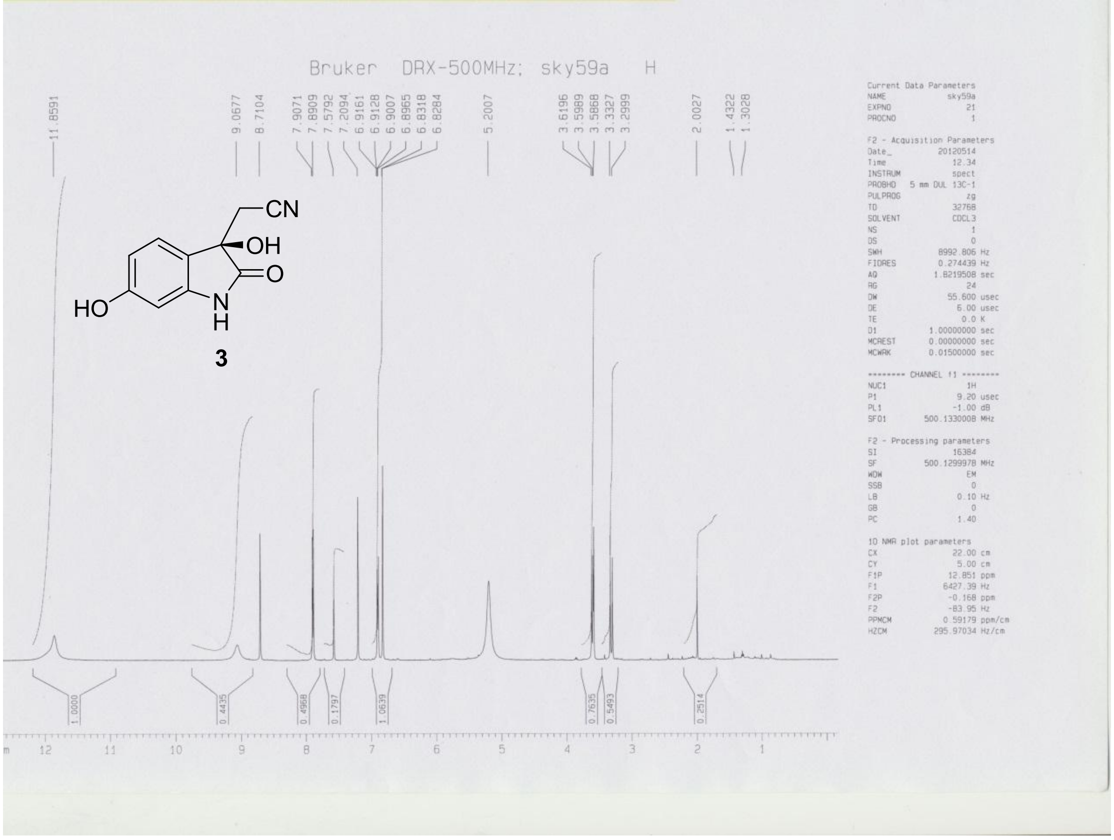

Figure 18S. ^{13}C NMR (125 MHz) spectrum of compound **3** in C_5D_5N.

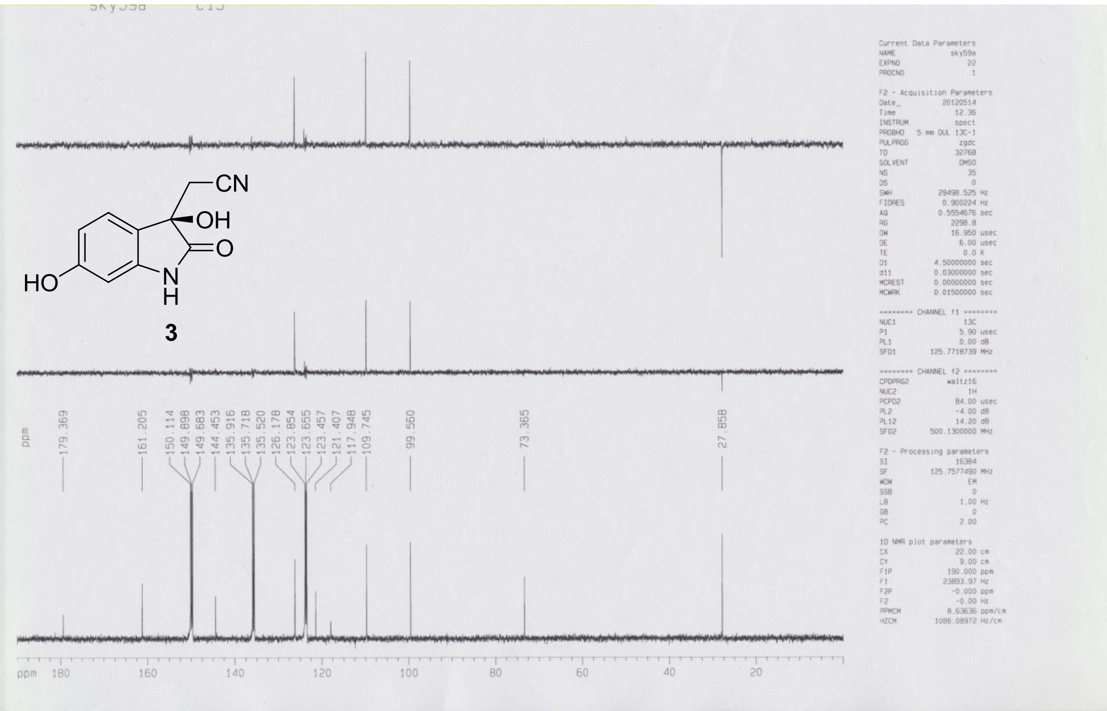

Figure 19S. HSQC (500 MHz) spectrum of compound **3** in C_5D_5N.

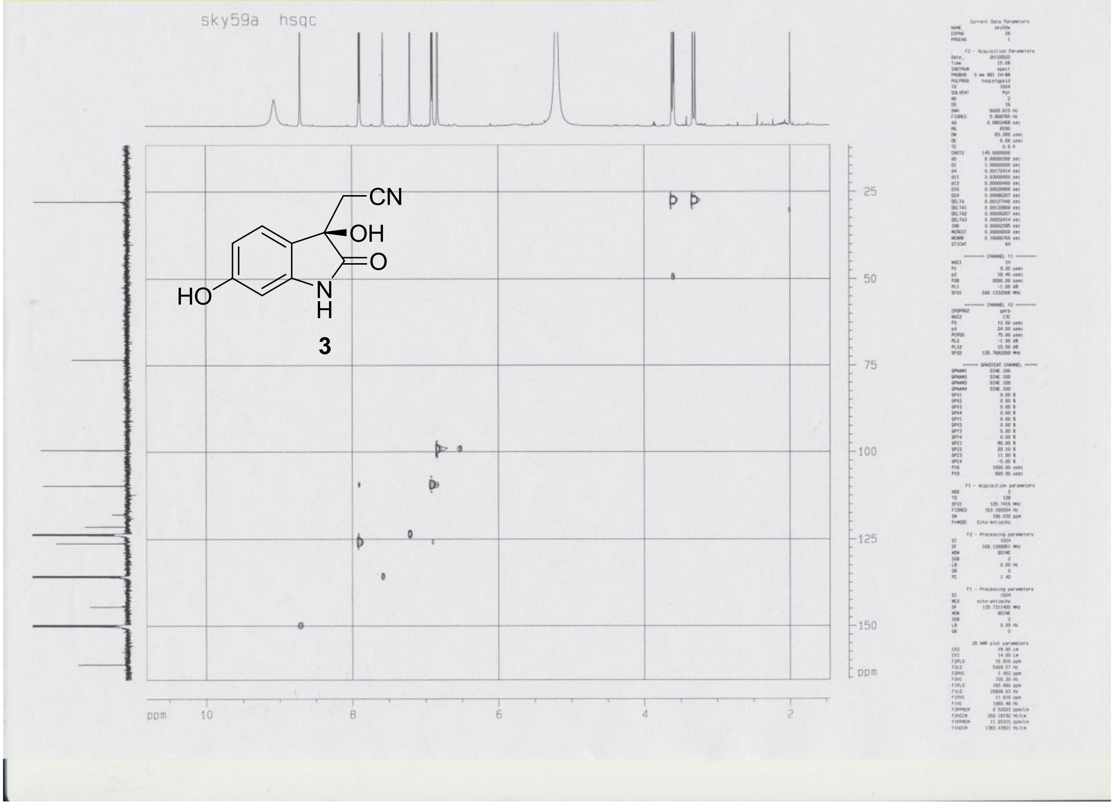

Figure 20S. HMBC (500 MHz) spectrum of compound **3** in C_5D_5N.

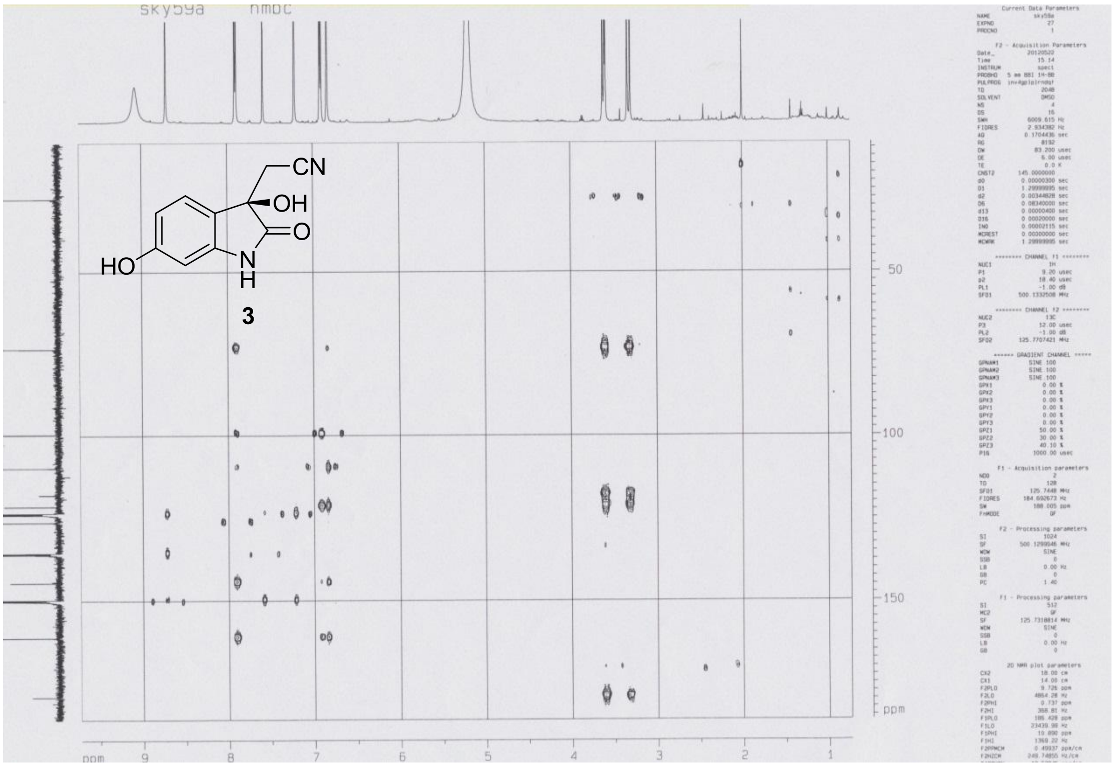

Figure 21S. ^{1}H-^{1}H COSY (500 MHz) spectrum of compound **3** in C_5D_5N.

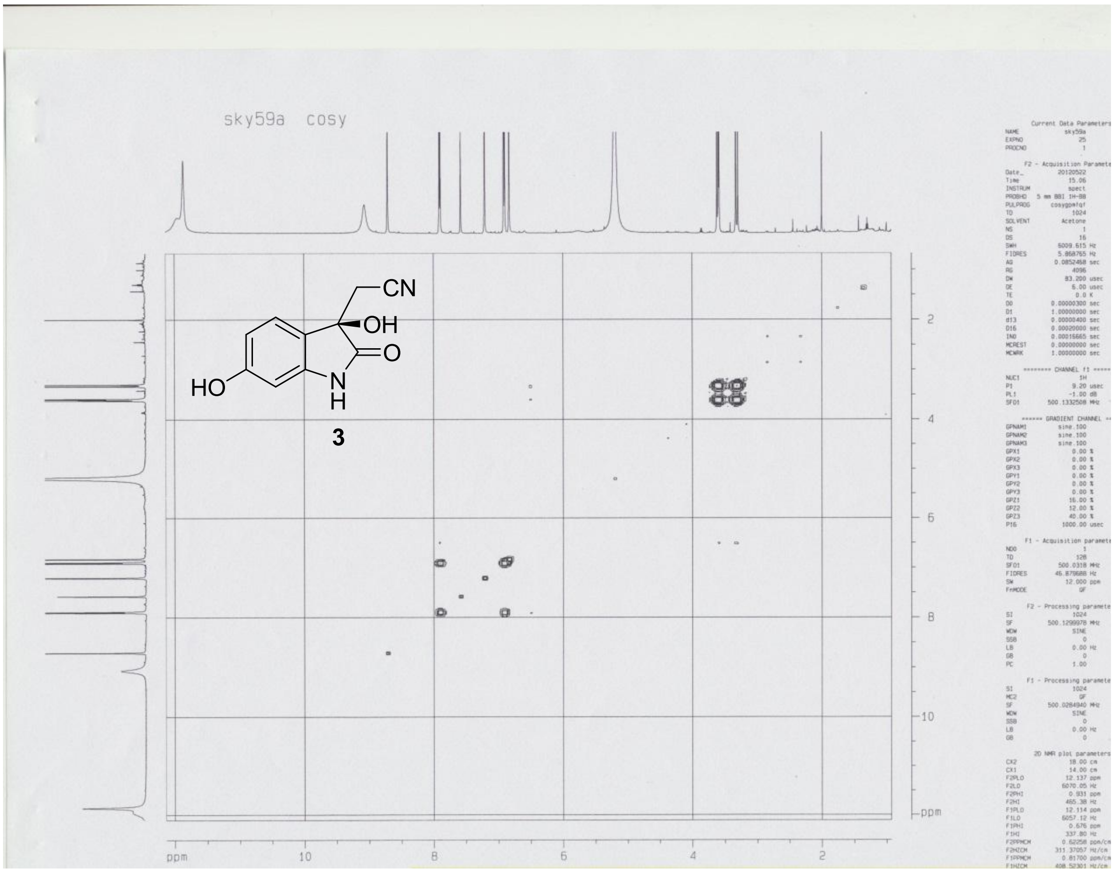

Figure 22S. ROESY (500 MHz) spectrum of compound **3** in C_5D_5N.

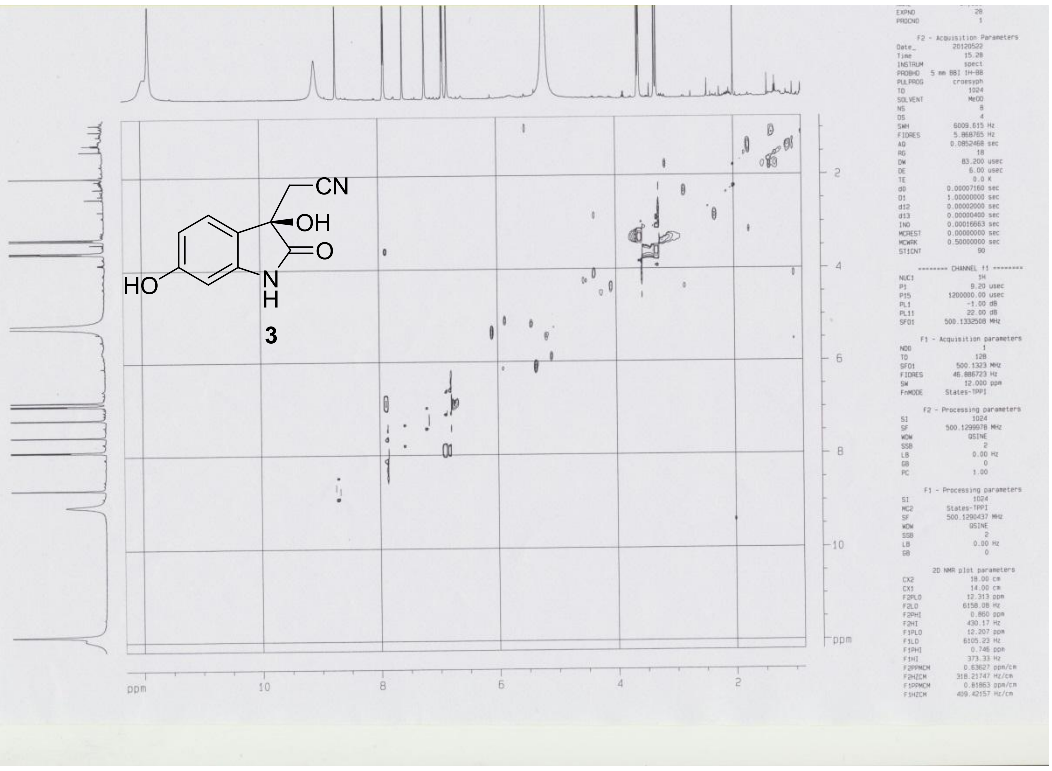

Figure 23S. HREIMS spectrum of compound **3**.

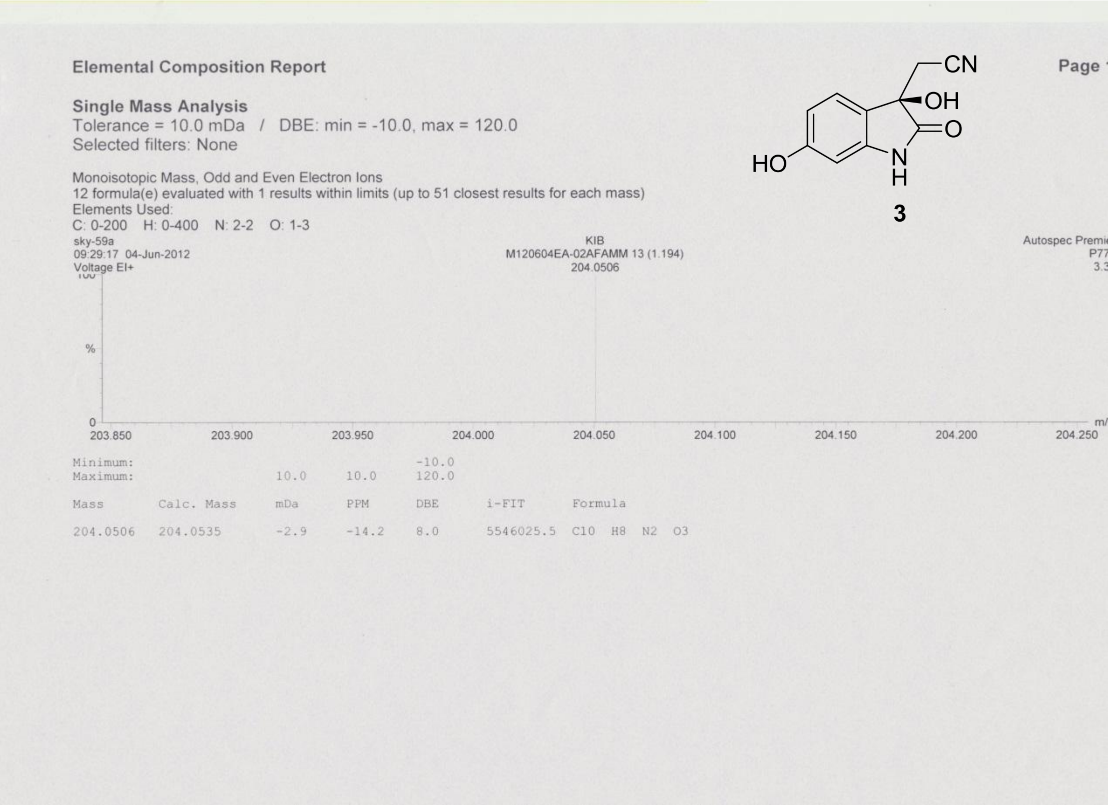

Figure 24S. CD spectrum of compound **3**.

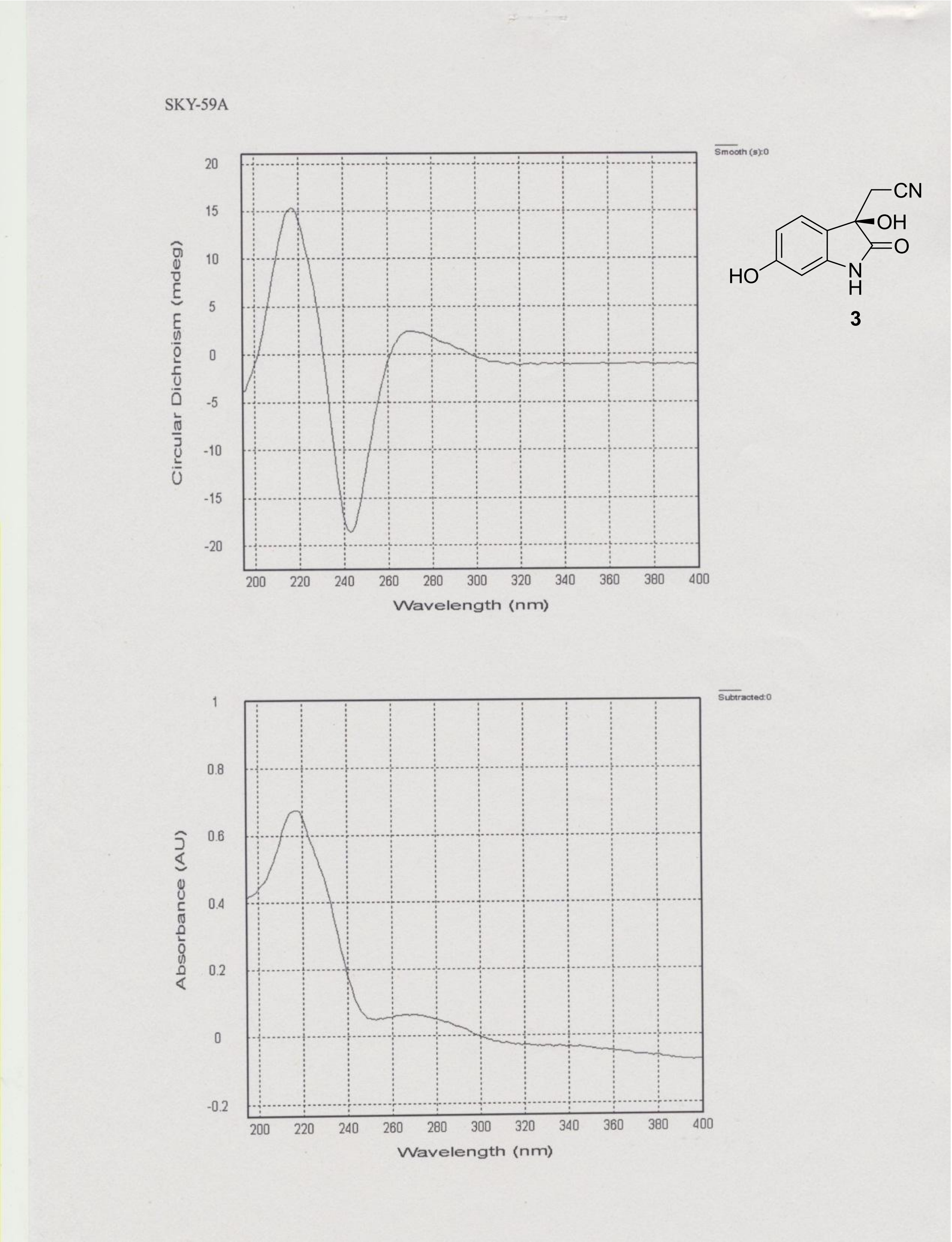

Figure 25S. ^{1}H NMR (600 MHz) spectrum of compound **4** in C_5D_5N.

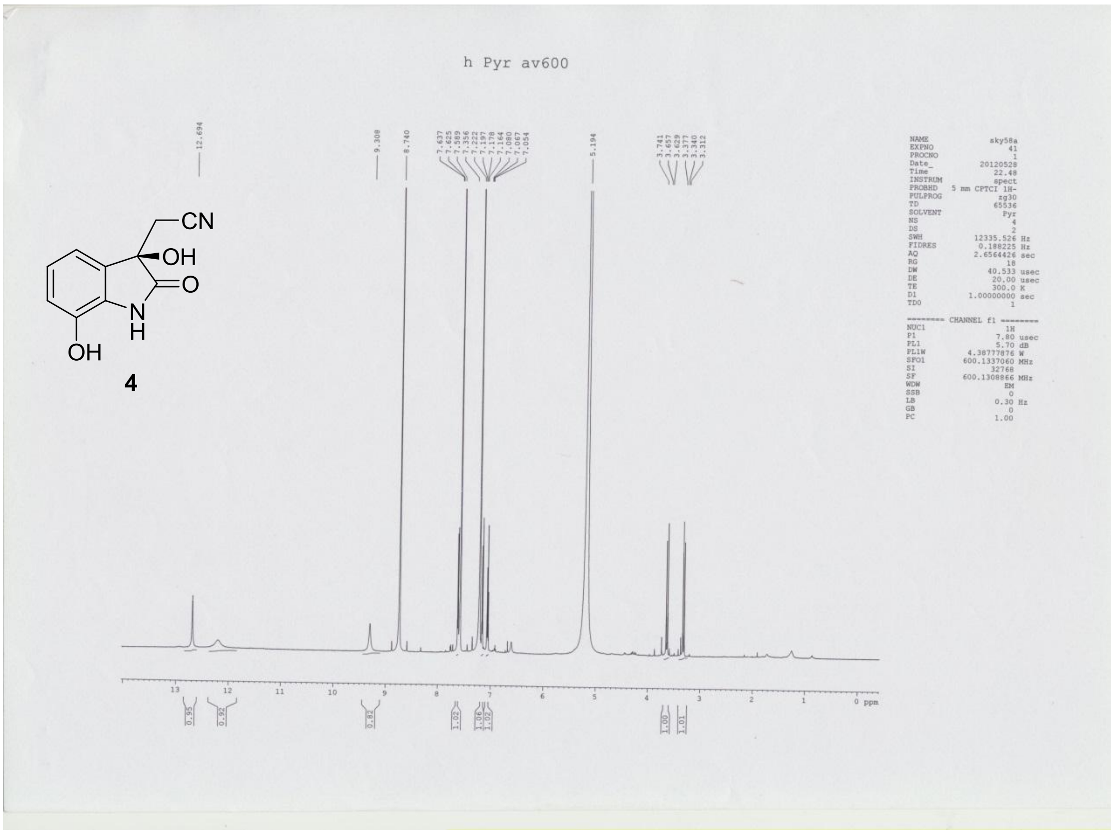

Figure 26S. ^{13}C NMR (150 MHz) spectrum of compound **4** in C_5D_5N.

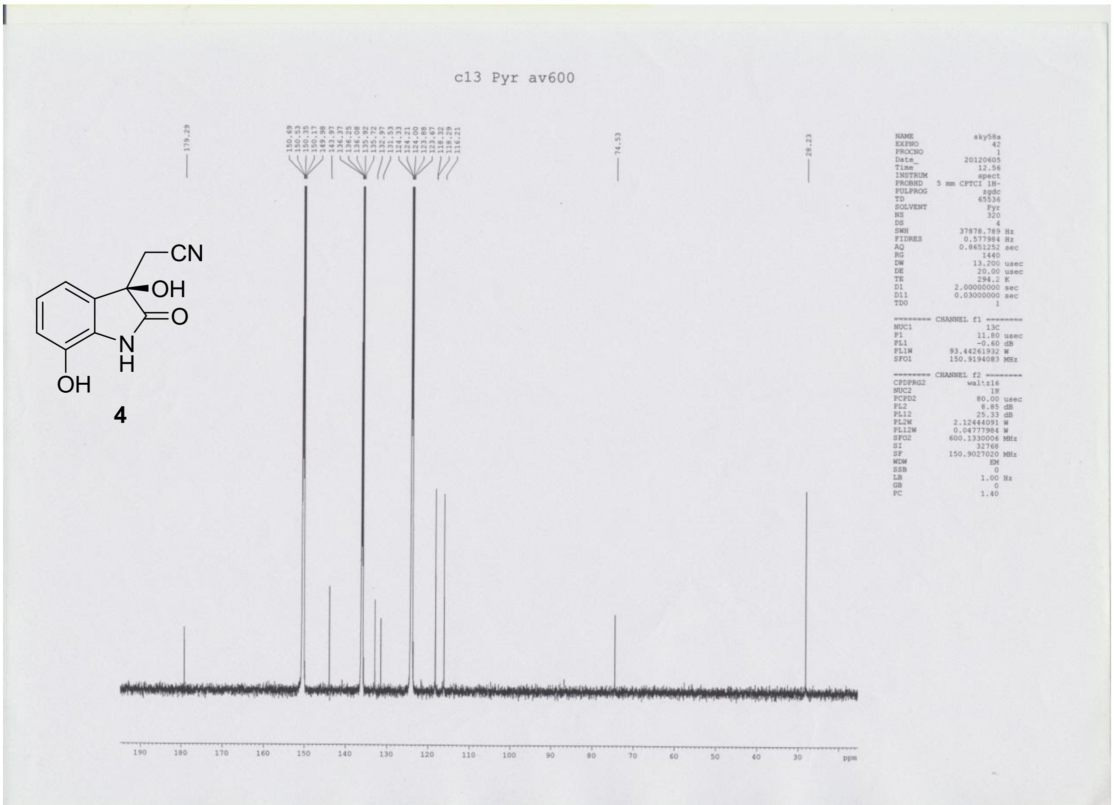

Figure 27S. HSQC (600 MHz) spectrum of compound **4** in C_5D_5N.

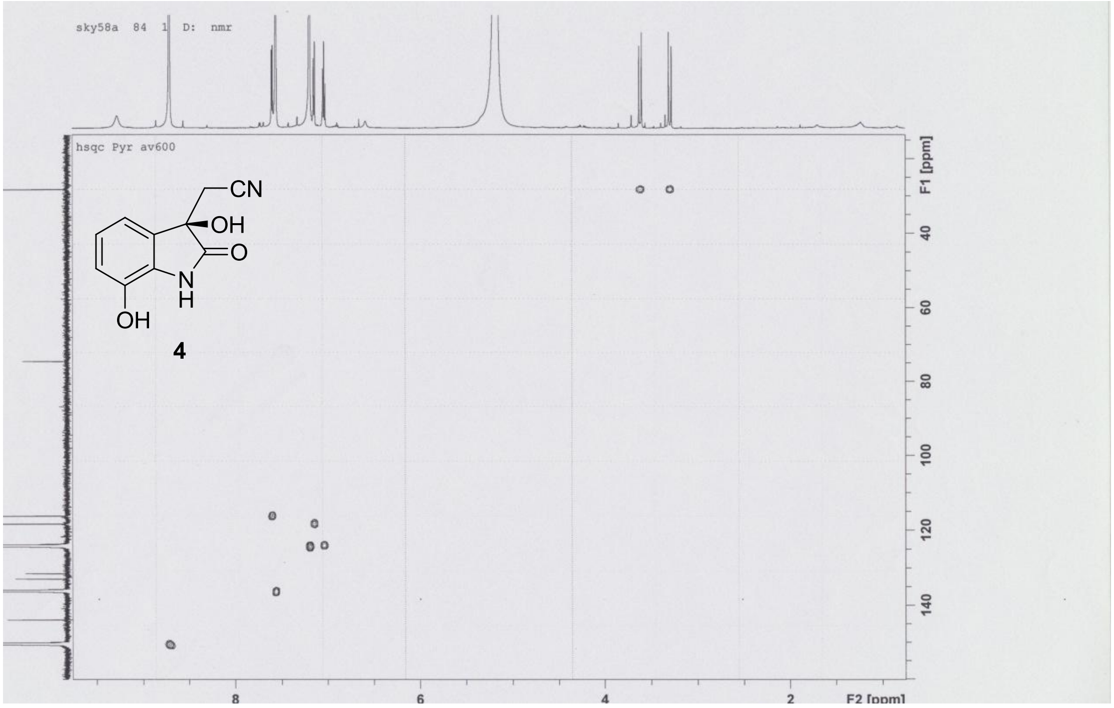

Figure 28S. HMBC (600 MHz) spectrum of compound **4** in C_5D_5N.

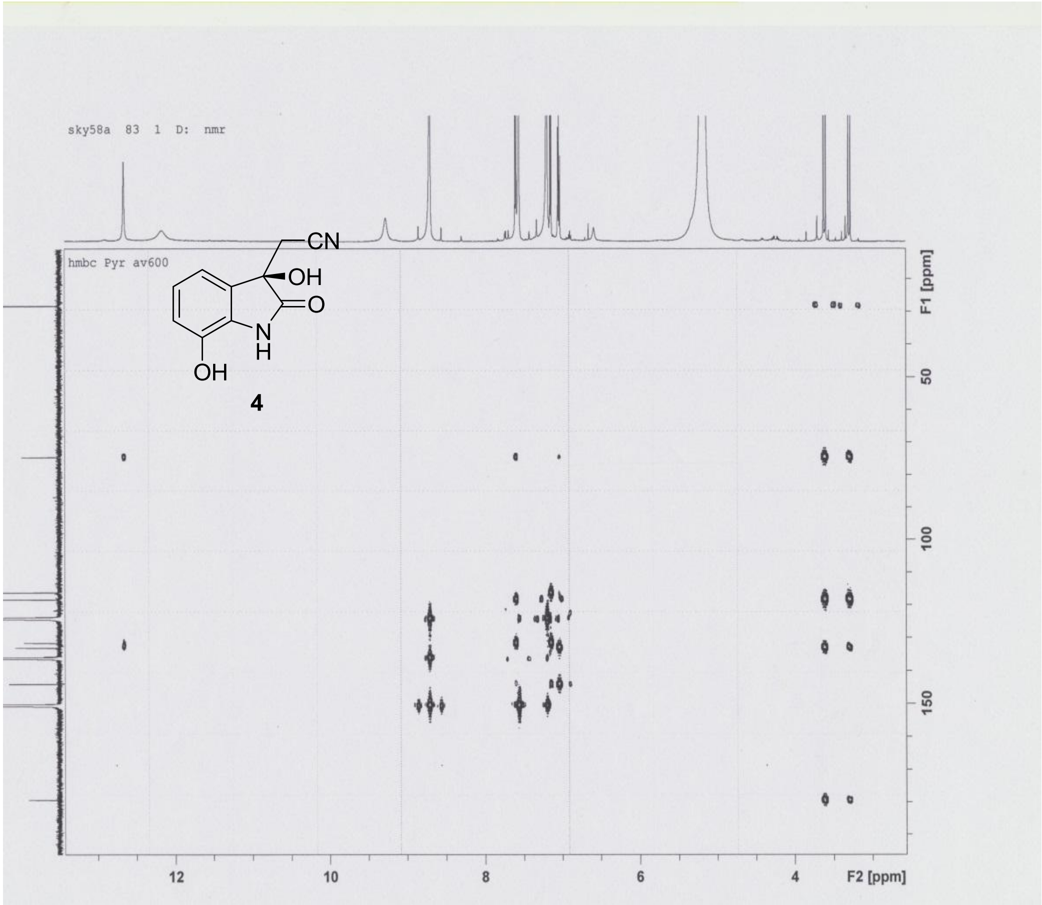

Figure 29S. ^{1}H-^{1}H COSY (600 MHz) spectrum of compound **4** in C_5D_5N.

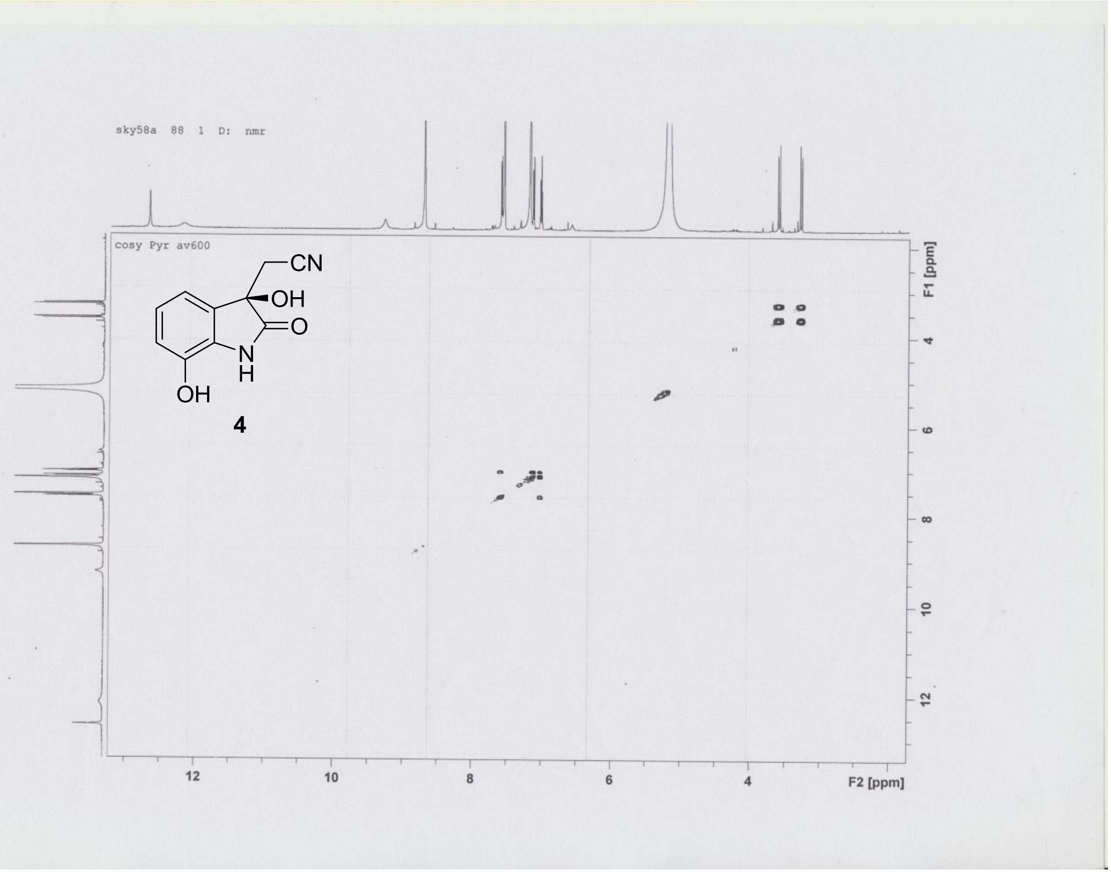

Figure 30S. ROESY (600 MHz) spectrum of compound **4** in C_5D_5N.

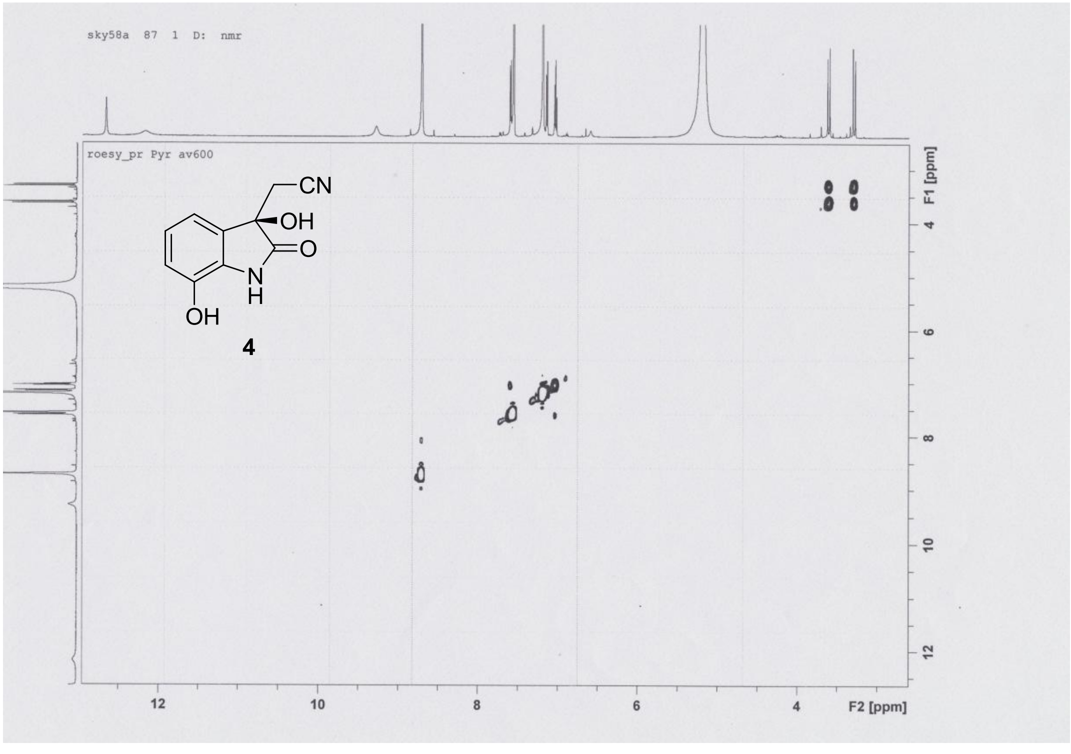

Figure 31S. HREIMS spectrum of compound **4**.

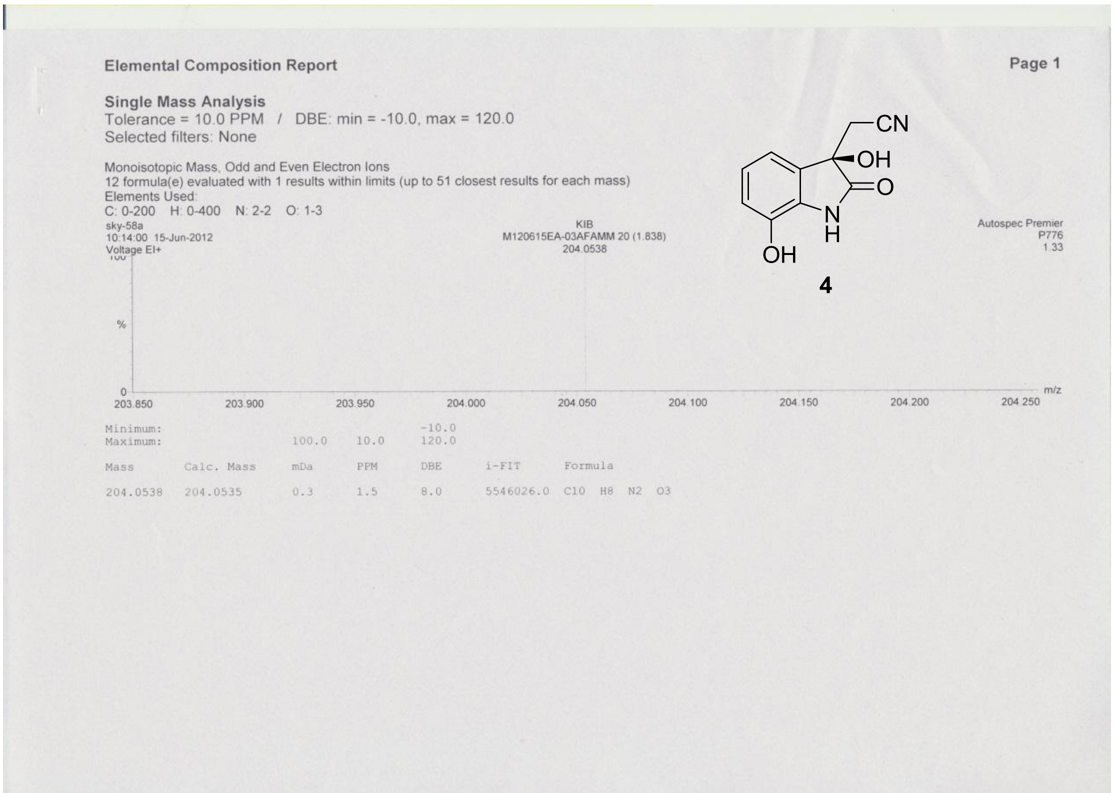

Elemental Composition Report

Page 1

Single Mass Analysis
Tolerance = 10.0 PPM / DBE: min = -10.0, max = 120.0
Selected filters: None

Monoisotopic Mass, Odd and Even Electron Ions
12 formula(e) evaluated with 1 results within limits (up to 51 closest results for each mass)
Elements Used:
C: 0-200 H: 0-400 N: 2-2 O: 1-3

Minimum:				-10.0		
Maximum:		100.0	10.0	120.0		
Mass	Calc. Mass	mDa	PPM	DBE	i-FIT	Formula
204.0538	204.0535	0.3	1.5	8.0	5546026.0	C10 H8 N2 O3

Figure 32S. CD spectrum of compound **4**.

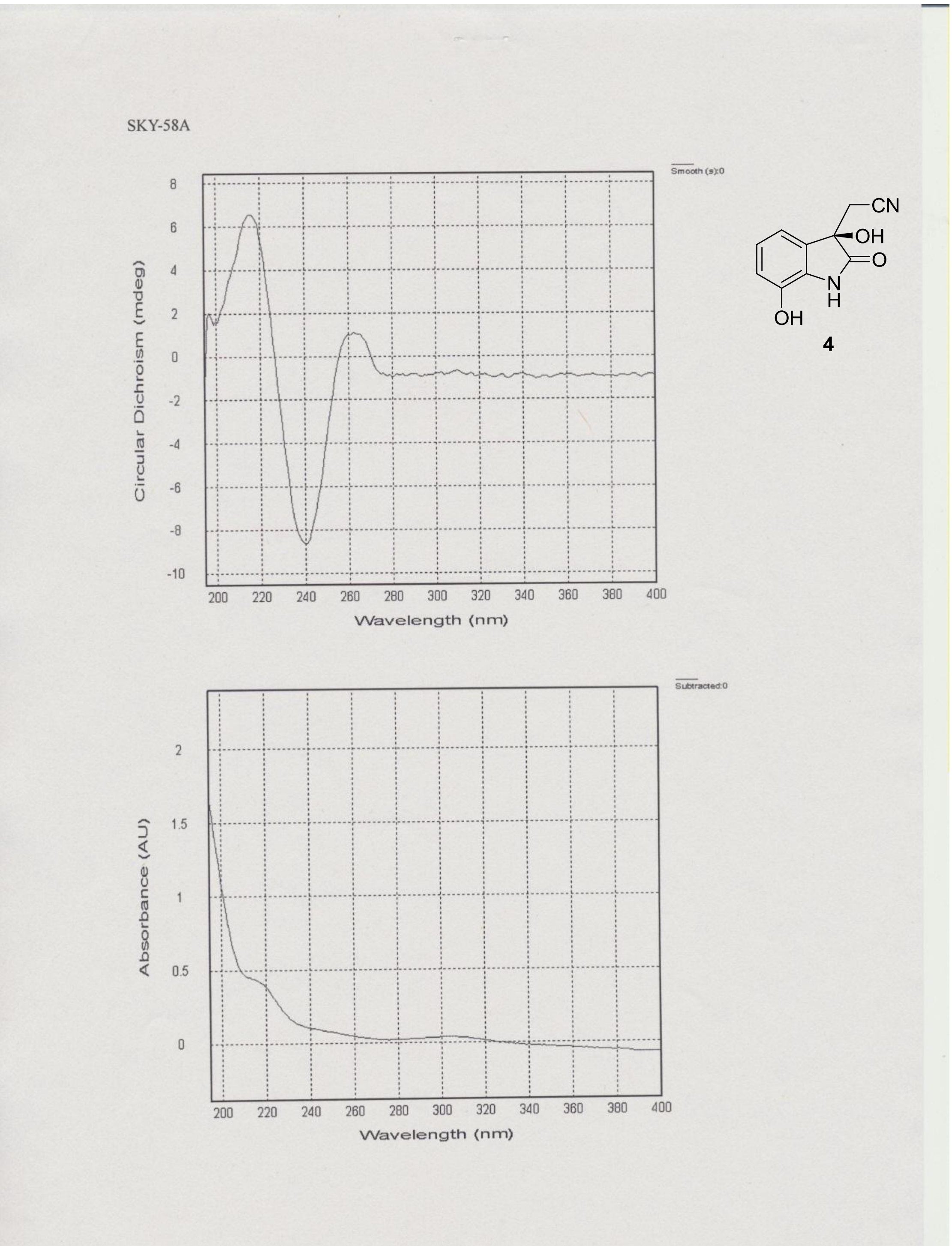

Supplement: Supplementary file 1 — Supplementary material, approximately 3.01 MB. [file 13659_2012_82_MOESM1_ESM.pdf]
